# On t-intuitionistic fuzzy graphs: a comprehensive analysis and application in poverty reduction

**DOI:** 10.1038/s41598-023-43922-0

**Published:** 2023-10-09

**Authors:** Asima Razzaque, Ibtisam Masmali, Laila Latif, Umer Shuaib, Abdul Razaq, Ghaliah Alhamzi, Saima Noor

**Affiliations:** 1 Department of Basic Sciences, Deanship of Preparatory Year, King Faisal University Al Ahsa, 31982 Al Hofuf, Saudi Arabia; 2 Department of Mathematics, College of Science, Jazan University, 45142 Jazan, Saudi Arabia; 3 Department of Mathematics, Government College University, Faisalabad, 38000 Pakistan; 4 Department of Mathematics, Division of Science and Technology, University of Education, Lahore, 54770 Pakistan; 5 Department of Mathematics and Statistics, College of Science, Imam Mohammad Ibn Saud Islamic University (IMSIU), 11564 Riyadh, Saudi Arabia

**Keywords:** Engineering, Mathematics and computing

## Abstract

This paper explains the idea of t-intuitionistic fuzzy graphs as a powerful way to analyze and display relationships that are difficult to understand. The article also illustrates the ability of t-intuitionistic fuzzy graphs to establish complex relationships with multiple factors or dimensions of a physical situation under consideration. Moreover, the fundamental set operations of t-intuitionistic fuzzy graphs are proposed. The notions of homomorphism and isomorphism of t-intuitionistic fuzzy graphs are also introduced. Furthermore, the paper highlights a practical application of the proposed technique in the context of poverty reduction within a specific society. By employing t-intuitionistic fuzzy graphs, the research demonstrates the potential to address the multifaceted nature of poverty, considering various contributing factors and their interdependencies. This application showcases the versatility and effectiveness of t-intuitionistic fuzzy graphs as a tool for decision-making and policy planning in complex societal issues.

## Introduction

Decision-making is essential to all aspects of existence. This also pertains to organizations. It is one of the most important factors in determining its success or failure. Every manager must make decisions throughout the management cycle, from planning to control. The level of a manager's success is influenced by the efficacy and caliber of his or her decisions. Without being able to make decisions, managers can't do their other jobs, like planning, organizing, supervising, controlling, and staffing. The decision-making process should be cumulative, consultative, and conducive to organizational growth. Fuzzy decision-making environments offer strategies for handling ambiguity and vagueness based on uncertainty. Ambiguity is a type of uncertainty in which it seems possible to choose more than one option from a list of options. It has been shown that fuzzy set theory (${\mathbb{FST}}$) is a good way to describe situations where the data are not clear or precise. A fuzzy set can handle this by giving each object in a set a certain amount of membership. In reality, however, a person may suppose that an object $"x"$ belongs to a set $A$ to a certain degree, yet not be entirely convinced. In other words, there may be hesitancy or uncertainty about “*x*” degree of participation in $A$. In ${\mathbb{FST}}$, there is no way to account for this uncertainty in membership degrees. Zadeh^1^ devised a mathematical method called fuzzy set theory (${\mathbb{FST}}$) to deal with information that comes from computational perception. This information is imprecise, unclear, ambiguous, vague, or doesn't have clear limits. Since its acceptance, this idea has been utilized in numerous technical and scientific domains. The ${\mathbb{FST}}$ has been used successfully in consumer electronics, control systems, image processing, knowledge-based systems, robotics, industrial automation, artificial intelligence, and consumer electronics. This theory has also been used in many areas of operations research, such as project management, decision theory, supply chain management, queue theory, and quality control. Mapari and Naidu^2^ studied some properties of ${{\mathbb{FS}}}$ and discussed their application Some introductory texts in this field were written by Kandel^3^, Klir and Yuan^4^, Mendel^5^, and Zimmermann^6^.

The intuitionistic fuzzy set (${\mathbb{IFS}}$) generalizes the fuzzy set because the indicator function of the ${{\mathbb{FS}}}$ is a particular case of the membership function and non-membership function of the ${\mathbb{IFS}}$. Atanassov^7^ introduced ${\mathbb{IFS}}$ as an extension of Zadeh's idea of fuzzy set, which itself is an extension of the traditional idea of a set. These sets are quite helpful in offering a flexible approach for elaborating the uncertainty and ambiguity inherent in decision making. De et al.^8^ presented the ${{\mathbb{IFS}}}$ operations and also demonstrated their various features. Several important aspects of the newly introduced operations on ${{\mathbb{IFS}}}$ were investigated in^9^. The ${{\mathbb{IFS}}}$ is an essential subject in fuzzy mathematics due to its vast range of real-world applications, including pattern recognition, machine learning, decision making, and market forecasting. Ejegwa et al.^10^ provided a clear and complete overview of various ${\mathbb{IFS}}$ models in real-world scenarios. Burillo et al.^11^ proposed the concept of intuitionistic fuzzy number (${{\mathbb{IFN}}}$). The ${\mathbb{IFN}}$ is a more general platform for communicating vague, incomplete, or contradictory information while solving multi-criteria decision-making problems and for expressing and reflecting evaluation information across multiple dimensions. Faizi et al.^12^ applied the concept of ${{\mathbb{IFS}}}$ in multi criteria group decision making. Dai et al.^13^ developed an intuitionistic fuzzy concept-oriented three-way decision model to tackle the ranking and classification problem in intuitionistic fuzzy multi-criteria contexts with the decision-maker's preference. Das et al.^14^ suggested a productive method for group MCDM based on intuitionistic multi-fuzzy set theory. These sets have been utilized in MCDM significantly more recently in^15–22^. In imaging applications, the enhancement of pictures with weak edges presents significant difficulties. Based on ${\mathbb{IFS}}$, Liu et al.^23^ developed a novel image enhancement technique. While color photographs give more information than grayscale images, segmenting color images is a task that is still in progress. The analysis of biomedical images is especially beneficial for numerous purposes. Bouchet et al.^24^ presented a method for the segmentation of leukocytes. This method combines the use of RGB color space, ${\mathbb{IFS}}$, and K-means clustering. Cagman and Karatas^25^ introduced the operation and application of intuitionistic fuzzy soft sets (${\mathbb{IFSS}}$). Ali et al.^26^ defined the aggregation operator for complex ${\mathbb{IFSS}}$ and developed their associated properties. Jabir et al.^27^ proposed algorithms based on a generalized ${\mathbb{IFSS}}$ and also showed the supremacy of the given methods. Bashir et al.^28^ introduced the possibility of ${\mathbb{IFSS}}$ and associated operations. The interval-valued ${\mathbb{IFSS}}$ theory was initiated by Jiang et al.^29^. A definition of a Hausdorff distance-based similarity measure between ${\mathbb{IFSS}}$ and its potential application in medical diagnosis were given in^30^. Deli and Karats^31^ established the concept of interval-valued intuitionistic fuzzy parameterized soft sets. They presented a decision-making method based on this notion in^32^. One of the most recent approaches to dealing with imprecision is the Pythagorean fuzzy set (PFS). These sets generalize ${\mathbb{IFS}}$ and have a wider range of uses, which inspires research into their applicability to the problem of career placement. Abdullah et al.^33^ depicted the Choquet integral operator based on PFSs. Fuzzy measures can be used to account for how parts of PFSs interact with each other.

A graph is a convenient method for describing data containing object relationships. Relationships are represented by edges and objects by vertices. It is commonly known that graphs are simple representations of relations. Graph theory provides a useful instrument for quantifying and simplifying the numerous moving pieces of dynamic systems. Mathematical chemistry examines the structure of molecules using mathematical methods. Molecular descriptors serve a key role in mathematical chemistry. As a field of study, chemical graph theory shows how chemistry, graph theory, and math are related. A molecular graph is a graph that represents the atoms and bonds of a compound via vertices and edges. With its vertices and edges, the graph makes it easy to see how different things are related to each other. Creating a “Fuzzy Graph Model” may be necessary to clarify the situation if there is any ambiguity in the description of objects or their relationships. They must deal with uncertain situations, and more information requires some high-potential tools. The graph is one such mathematical tool which effectively deals with extensive data. Fuzzy graph is a tool that needs to be used when uncertain factors exist. Rosenfeld^34^ took the first step into the field of fuzzy graph. Mordeson and Chang-Shyh^35^ discussed certain fuzzy graph operations. Bhattacharya^36^ proved several graph theoretic results for fuzzy graph. Bhutani^37^ worked on automorphisms of fuzzy graph. The fuzzy graph is used in a wide range of scientific and engineering fields, such as broadcast communications, production, social networks, artificial intelligence, data hypotheses, and neural systems. The study of fuzzy graph led many researchers to contribute in this fields. Pathinathan et al.^38^ initiated the idea of hesitant fuzzy graph. Javaid et al.^39^ proposed numerous operations on hesitant fuzzy graphs. Moreover, Akram and Saira^40^ introduced the notion of fuzzy soft graphs and they also presented the applications of fuzzy soft graphs in social and road networks^41^. Ali et al.^42^ initiated the complex q-rung orthopair fuzzy planar graph theory. Kifayat et al.^43^ explored the ideas of complex q-rung orthopair fuzzy k-competition, complex q-rung orthopair fuzzy p-competition, and complex q-rung orthopair fuzzy neighborhoods. Fuzzy graphs have been applied to many practical situations like optimization problems^44,45^, clustering^46^ and social networks^47^. Intuitionistic fuzzy graphs (${\mathbb{IFG}}$) provide a more accurate representation of human thinking and decision-making processes. Individuals frequently need clarification about the precise acceptance or rejection of an element inside a particular set. The ${\mathbb{IFG}}$ and intuitionistic fuzzy relations were introduced by Shannon and Atanassov^48^ and they also looked into some of their characteristics^49^. Karunambigai and Atanassov^50^ studied operations on ${\mathbb{IFG}}$. Gani and Begum^51^ discussed the size, order and degree of ${\mathbb{IFG}}$. Sundas and Akram^52^ described the application of an intuitionistic fuzzy soft graph to a problem involving decision-making. Yaqoob et al.^53^ developed the complex intuitionistic fuzzy graph theory. Abida and Faryal^54^ classified the fundamental operations as direct, semi-strong, strong, and modular products for complex intuitionistic fuzzy graphs. Nandhinii and Amsaveni^55^ proposed a bipolar complex intuitionistic fuzzy graph. Furthermore, the literature has extensively examined many principles and applications of ${\mathbb{IFG}}$ and their expansions, as evidenced by the works cited in references^56–59^. The theory of t-${\mathbb{IFS}}$ was initiated by Sharma in^60^.

The t-${\mathbb{IFS}}$ has shown advantages in handling vagueness and uncertainty compared to intuitionistic fuzzy set. It's a good strategy because it gives a flexible way to deal with the uncertainty and ambiguity that come with making decisions. The t-Intuitionistic fuzzy models are becoming more useful because they try to close the gap between traditional numerical models used in engineering and the sciences and symbolic models used in expert systems. The theory of ${\mathbb{IFG}}$ serves as a valuable tool for delineating and clarifying complex and indeterminate matters that arise in practical contexts. This phenomenon can be attributed to its ability to effectively communicate the inherent characteristics of unpredictability, complexity, imprecision, and uncertainty connected with the things encompassed inside these sets. However, it is necessary to rewrite these approaches using specific numerical values to effectively handle the practical concerns related to membership and non-membership functions. To overcome this constraint, we presented the concept of a t-${\mathbb{IFG}}$, which utilizes linear t-norm and t-conorm operators. The need for a systematic and adaptable methodology to effectively handle ambiguity and enable decision-making under the guidance of pre-established criteria led to the adoption of the t-${\mathbb{IFG}}$. In this context, the utilization of the parameter ‘t’ facilitates the simplification of the procedure by specifying particular criteria for identifying the degree of membership or non-membership. In many practical scenarios, it becomes imperative to make judgments contingent on different levels of confidence. Introducing the parameter ‘t’ in the t-${\mathbb{IFG}}$ aims to overcome the constraints of the ${\mathbb{IFG}}$. This parameter offers precise control over stringency, enhances customization, allows for separate thresholds for decision-making, enhances flexibility, and reduces ambiguity. The benefits above render the t-${\mathbb{IFG}}$ a very effective technique for depicting uncertainty and facilitating well-informed decision-making in contexts that necessitate a tailored and regulated approach to uncertainty management. The t-${\mathbb{IFG}}$ facilitates the understanding and manipulation of complex decision environments in situations where traditional ${\mathbb{IFG}}$ is insufficient. The role of complicated, ambiguous interactions is essential in the context of decision-making challenges. These graphs thoroughly describe the complex interplay between input and output variables, offering decision-makers powerful tools for analyzing and assessing different choices. Complicated fuzzy connections allow decision-makers to determine options comprehensively and systematically by considering various criteria and their interdependencies. This facilitates a holistic approach to addresses complex decision-making difficulties. The intricate technique represents a significant advancement in decision-making, particularly in situations characterized by membership, non-membership, and parameter t. It signifies a break from the limitations imposed by binary logic and paves the way for enhanced accuracy in decision-making processes.

The subsequent motivation for organizing research is presented:

- The primary motivation for using t-IFG is their capacity to effectively handle intricate uncertainty scenarios characterized by hesitant and fluctuating interactions between elements.
- By including the "t" parameter, these graphs offer a framework for assessing and modeling diverse levels of uncertainty and confidence in connections.
- Incorporating t-norms and t-conorms provides a method for handling the combination and disjunction of uncertain information, designed explicitly for decision-making situations involving a wide range of inputs and outcomes in the real world.
- This approach is employed in several fields, like decision analysis, risk assessment, and systems optimization, where the objective is to achieve a trade-off between unknown connections and practical value.

Integrating intuitionistic fuzzy logic, graph theory, and the parameter "t" gives rise to t-intuitionistic fuzzy graphs, which present a novel methodology. The following are the novelties of the present work:

- The parameter denoted as "t" represents a threshold that indicates reluctance, enabling the creation of a new and organized representation of unclear connections.
- Incorporating the "t" parameter can enhance the depiction of relationships, wherein the selection of edges and nodes is dependent upon keeping to a defined confidence level.
- This methodology would provide a more precise differentiation between robust and delicate associations, enabling more systematic handling of ambiguity.
- A t-${\mathbb{IFG}}$ allows for incorporating multi-layered analysis, wherein different graph levels are associated with various parameter values “t”. By employing this approach, it would be possible to thoroughly examine the interconnections within the graph, taking into account different degrees of certainty. It facilitates a deeper understanding of the fundamental framework.

Our primary goals for this article are to make the following contributions:

- Propose the idea of the t-${\mathbb{IFG}}$. This phenomenon is advantageous in that it offers a flexible paradigm for describing the uncertainty and ambiguity inherent in decision-making. Moreover, it plays a significant role in various disciplines such as computer science, economics, chemistry, medicine, and engineering.
- Explore various set theoretical operations of t-${\mathbb{IFG}}$ and prove many key properties of the newly defined operations. These operations enable the integration of information, the exploration of connections, and the facilitation of informed decision-making across various application domains.
- Introduce the notions of homomorphism and isomorphism of t-${\mathbb{IFG}}$ and demonstrate many newly defined key properties. This notion is used to improve the comfort of conducting comparative analysis and transmitting data in scenarios that include graph topologies that are unsure and hesitant.
- Initiate the idea of the complement of a t-${\mathbb{IFG}}$ and prove many vital properties of this notion. This notion of ambiguity exposes inverse relationships that may not be directly evident in the original graph. The applications of this technology encompass error detection, system verification, and decision analysis.
- Identify the critical factors for reducing poverty in a certain society using the newly defined technique. This technique will help reduce poverty by improving representation, identifying susceptible groups, allocating resources, tracking, and evaluating progress, and formulating well-considered policies.
- Explores the complexity and uncertainties of poverty, leading to an assessment of the causes, development, and impacts.

Following a brief discussion of the t-${\mathbb{IFG}}$, the rest of the paper is structured as follows: In “Preliminaries” section, some fundamental definitions are provided to help the reader to comprehend the originality of the work presented in this article. In "t-Intuitionistic Fuzzy Graph" section, the notion of t-${\mathbb{IFG}}$ is introduced and various fundamental characteristics of this phenomenon are investigated. In "Operations on t-intuitionistic fuzzy graph" section, various set theoretical operations of t-${\mathbb{IFG}}$ are explored and graphical representations of these operations are demonstrated. In “Isomorphism of t-intuitionistic fuzzy graphs” section, the concepts of homomorphisms and isomorphisms of t-${\mathbb{IFG}}$ are established. In “Complement of t-intuitionistic fuzzy graph” section, the idea of complement of t-${\mathbb{IFG}}$ is defined and many important key features of this notion are explored. In “Application of t-intuitionistic fuzzy graph” section, the newly defined strategy is applied to design a mechanism for the reduction of poverty in a certain society. Finally, some comparative analysis and concrete conclusions about the paper are summarized in “Comparative analysis” and “Conclusion” sections respectively.

The list of abbreviations used in this article is shown in the table below.

**Table:** 

${\mathbb{IFS}}$	Intuitionistic fuzzy set	${\mathbb{IFSS}}$	Intuitionistic fuzzy soft sets
${\mathbb{IFN}}$	Intuitionistic fuzzy number	MCDM	Multi-criteria decision making
${\mathbb{IFG}}$	Intuitionistic fuzzy graph	t-${\mathbb{IFS}}$	t-intuitionistic fuzzy set
PFS	Pythagorean fuzzy set	t-${\mathbb{IFG}}$	t-intuitionistic fuzzy graph

## Preliminaries

The fundamental concepts and definitions of t-${\mathbb{IFS}}$ are explained in this section.

### Definition 1^7^

 An ${\mathbb{IFS}}$ $\mathfrak{B}$ of a universe ${\mathbb{U}}$ of the form: $\mathfrak{B}= \{ < {{u}}_{1}, {\mu }_{\mathfrak{B}}\left({{u}}_{1}\right), {\sigma }_{\mathfrak{B}}\left({{u}}_{1}\right)>:{{u}}_{1}\in {\mathbb{U}}\},$ where $\mu_{{\mathfrak{B}}}$ and $\sigma_{{\mathfrak{B}}}$ are the functions from universe ${\mathbb{U}}$ to $\left[ {0, 1} \right],$ respectively, the membership and non-membership of an element $u_{1}$ of the universe ${\mathbb{U}}$ respectively. These functions must satisfy the following condition: $0 \le$ $\mu_{{\mathfrak{B}}} \left( {u_{1} } \right) + \sigma_{{\mathfrak{B}}} \left( {u_{1} } \right) \le 1.$

### Definition 2^60^

 Let ${\mathfrak{B}}$ be an ${\mathbb{IFS}}$ of a universal set ${\mathbb{U}}$ and $t \in \left[ {0,1} \right].$ The ${\mathbb{IFS}}$ ${\mathfrak{B}}_{t}$ of ${\mathbb{U}}$ is called a t-intuitionistic fuzzy set (t-${\mathbb{IFS}}$) and is defined as: $\mu_{{{\mathfrak{B}}_{t} }} \left( {u_{1} } \right) = min\{ \mu_{{\mathfrak{B}}} \left( {u_{1} } \right),t\}$ and $\it \sigma_{{{\mathfrak{B}}_{t} }} \left( {u_{1} } \right) = \max \left\{ {\sigma_{{\mathfrak{B}}} \left( {u_{1} } \right), 1 - t} \right\},\forall u_{1} \in {\mathbb{U}}$. The value of $\tau \left( {u_{1} } \right) = 1 - \left( {\mu_{{A_{t} }} \left( {u_{1} } \right) + \sigma_{{A_{t} }} \left( {u_{1} } \right)} \right)$ is called the degree of hesitancy. The t-${\mathbb{IFS}}$ is of the form: ${\mathfrak{B}}_{t} = \left\{ {\left( {u_{1} ,\mu_{{{\mathfrak{B}}_{t} }} \left( {u_{1} } \right),\sigma_{{{\mathfrak{B}}_{t} }} \left( {u_{1} } \right)} \right):u_{1} \in {\mathbb{U}}} \right\},$ where $\mu_{{{\mathfrak{B}}_{t} }}$ and $\sigma_{{{\mathfrak{B}}_{t} }}$ are functions that assign degrees of membership and non-membership, respectively. Moreover, the functions $\mu_{{{\mathfrak{B}}_{t} }}$ and $\sigma_{{{\mathfrak{B}}_{t} }}$ satisfy the condition: $0 \le \mu_{{{\mathfrak{B}}_{t} }} \left( {u_{1} } \right) + \sigma_{{{\mathfrak{B}}_{t} }} \left( {u_{1} } \right) \le 1.$

### Definition 3^48^

 Let ${{\mathbb{G}}^{\prime}} = \langle{\mathbb{V}},{\mathbb{E}}\rangle$ be a simple graph. A pair ${\mathcal{G}} = \langle{\mathcal{A}},{\mathcal{B}}\rangle$ is said to be an intuitionistic fuzzy graph (${\mathbb{IFG}}$) on graph ${{\mathbb{G}}^{\prime}},$ where ${\mathcal{A}} = \left\{ { \left\langle { u_{i} , \mu_{{\mathcal{A}}} \left( {u_{i} } \right), \sigma_{{\mathcal{A}}} \left( {u_{i} } \right)} \right\rangle :u_{i} \in {\mathbb{V}}} \right\}$ is an ${\mathbb{IFS}}$ on ${\mathbb{V}}$ and $\mathcal{B}=\{ < {{u}}_{i}, {\mu }_{\mathcal{B}}\left({{u}}_{i},{{u}}_{j}\right), {\sigma }_{\mathcal{B}}\left({{u}}_{i},{{u}}_{j}\right)>:\left({{u}}_{i},{{u}}_{j}\right)\in {\mathbb{E}}\}$ is an ${\mathbb{IFS}}$ on ${\mathbb{E}} \subseteq {\mathbb{V}} \times {\mathbb{V}}$ such that for every edge $\left( {u_{i} ,u_{j} } \right) \in {\mathbb{E}}$.

$$\begin{aligned} & \mu_{{\mathcal{B}}} \left( {u_{i} ,u_{j} } \right) \le min\left\{ {\mu_{{\mathcal{A}}} \left( {u_{i} } \right),\mu_{{\mathcal{A}}} \left( {u_{j} } \right)} \right\} \\ & \sigma_{{\mathcal{B}}} \left( {u_{i} ,u_{j} } \right) \le max\left\{ {\sigma_{{\mathcal{A}}} \left( {u_{i} } \right),\sigma_{{\mathcal{A}}} \left( {u_{j} } \right)} \right\} \\ \end{aligned}$$

Satisfy the conditions: $0\le {\mu }_{\mathcal{A}}\left({{u}}_{i}\right)+{\sigma }_{\mathcal{A}}\left({{u}}_{i}\right)\le 1$ and $0\le {\mu }_{\mathcal{B}}\left({{u}}_{i},{{u}}_{j}\right)+{\sigma }_{\mathcal{B}}\left({{u}}_{i},{{u}}_{j}\right)\le 1.$

### Definition 4^51^

 The order of ${\mathbb{IFG}}$ $\mathcal{G}$ is specified by:

$${\mathcal{O}}\left( {\mathcal{G}} \right) = \left( {\mathop \sum \limits_{{u_{1} \in {\mathbb{V}}}} \mu_{{\mathcal{A}}} \left( {u_{1} } \right),\mathop \sum \limits_{{u_{1} \in {\mathbb{V}}}} \sigma_{{\mathcal{A}}} \left( {u_{1} } \right)} \right)$$

### Definition 5^51^

 The degree of a vertex ${{u}}_{1}$ in ${\mathbb{IFG}}$ $\mathcal{G}$ is given by:

$$deg_{{\mathcal{G}}} \left( {u_{1} } \right) = \left( {deg_{{\mu_{{\mathcal{B}}} }} \left( {u_{1} } \right),deg_{{\sigma_{{\mathcal{B}}} }} \left( {u_{1} } \right)} \right) = \left( {\mathop \sum \limits_{{\left( {u_{1} ,u_{2} } \right) \in {\mathbb{E}}}} \mu_{{\mathcal{B}}} \left( {u_{1} ,u_{2} } \right),\mathop \sum \limits_{{\left( {u_{1} ,u_{2} } \right) \in {\mathbb{E}}}} \sigma_{{\mathcal{B}}} \left( {u_{1} ,u_{2} } \right)} \right)$$

## t-intuitionistic fuzzy graph

This section defines a t-intuitionistic fuzzy graph and explores various fundamental properties of this phenomenon.

### Definition 6

Let ${\mathcal{G}} = \left\langle {{\mathcal{A}},{ \mathcal{B}}} \right\rangle$ be an intuitionistic fuzzy graph (${\mathbb{IFG}}$) on a simple graph ${{\mathbb{G}}^{\prime}} = \left\langle {{\mathbb{V}},{\mathbb{E}}} \right\rangle$. An ${\mathbb{IFG}}$ ${\mathcal{G}}$ is called a t-intuitionistic fuzzy graph (t-${\mathbb{IFG}}$) is denoted by ${\mathcal{G}}_{t} = \left\langle {{\mathcal{A}}_{t} ,{\mathcal{B}}_{t} } \right\rangle ,$ where ${\mathcal{A}}_{t} = \left\{ {\left( {u_{i} , \mu_{{{\mathcal{A}}_{t} }} \left( {u_{i} } \right),\sigma_{{{\mathcal{A}}_{t} }} \left( {u_{i} } \right)} \right):u_{i} \in {\mathbb{V}}} \right\}$ is a t-${\mathbb{IFS}}$ on ${\mathbb{V}}$ and ${\mathcal{B}}_{t} = \left\{ {\left( {\left( {u_{i} ,u_{j} } \right), \mu_{{{\mathcal{B}}_{t} }} \left( {u_{i} ,u_{j} } \right),\sigma_{{{\mathcal{B}}_{t} }} \left( {u_{i} ,u_{j} } \right)} \right):\left( {u_{i} ,u_{j} } \right) \in {\mathbb{E}}} \right\}$ is a t-${\mathbb{IFS}}$ on ${\mathbb{E}} \subseteq {\mathbb{V}} \times {\mathbb{V}},$ such that for every edge $\left( {u_{i} ,u_{j} } \right) \in {\mathbb{E}}$.

$$\begin{aligned} & \mu_{{{\mathcal{B}}_{t} }} \left( {u_{i} ,u_{j} } \right) \le min\left\{ {\mu_{{{\mathcal{A}}_{t} }} \left( {u_{i} ,} \right),\mu_{{{\mathcal{A}}_{t} }} \left( {u_{j} } \right)} \right\} \\ & \sigma_{{{\mathcal{B}}_{t} }} \left( {u_{i} ,u_{j} } \right) \le max\left\{ {\sigma_{{{\mathcal{A}}_{t} }} \left( {u_{i} } \right),\sigma_{{{\mathcal{A}}_{t} }} \left( {u_{j} } \right)} \right\} \\ \end{aligned}$$

Satisfy the conditions: $0\le {\mu }_{{\mathcal{A}}_{t}}\left({{u}}_{i}\right)+{\sigma }_{{\mathcal{A}}_{t}}\left({{u}}_{i}\right)\le 1$ and $0\le {\mu }_{{\mathcal{B}}_{t}}\left({{u}}_{i},{{u}}_{j}\right)+{\sigma }_{{\mathcal{B}}_{t}}\left({{u}}_{i},{{u}}_{j}\right)\le 1.$

Here ${\mu }_{{\mathcal{A}}_{t}}\left({{u}}_{i}\right)$ and ${\sigma }_{{\mathcal{A}}_{t}}\left({{u}}_{i}\right)$ represents the membership and non-membership degrees of nodes ${{u}}_{i}\in {\mathbb{V}}.$ The terms ${\mu }_{{\mathcal{B}}_{t}}\left({{u}}_{i},{{u}}_{j}\right)$ and ${\sigma }_{{\mathcal{B}}_{t}}\left({{u}}_{i},{{u}}_{j}\right)$ represents the membership and non-membership degrees of edges $({{u}}_{i},{{u}}_{j})\in {\mathbb{E}},$ respectively.

### Example 1

Consider a graph ${\mathbb{G}}^{\prime}=\langle {\mathbb{V}},{\mathbb{E}}\rangle$ such that.

$${\mathbb{V}} = \left\{ {a,b,c,d,e,f} \right\}\quad {\text{and}}\quad {\mathbb{E}} = \left\{ {ab,ac,af,bc,cd,ce,de,ef} \right\}.$$

The ${\mathbb{IFS}}$ ${\mathcal{A}}$ of ${\mathbb{V}}$ is given by:

$${\mathcal{A}} = \left\{ {\left( {a,0.8,0.2} \right),\left( {b,0.9,0.1} \right),\left( {c,0.5,0.4} \right),\left( {d,0.7,0.2} \right),\left( {e,0.6,0.4} \right),\left( {f,0.8,0.1} \right)} \right\}$$

The ${\mathbb{IFS}}$ ${\mathcal{B}}$ of ${\mathbb{E}}$ is given by:

$${\mathcal{B}} = \left\{ {\begin{array}{*{20}c} {\left( {ab,0.3,0.3} \right),\left( {ac,0.5,0.4} \right),\left( {af,0.6,0.3} \right),\left( {bc,0.4,0.3} \right),\left( {cd,0.5,0.3} \right),} \\ {\left( {ce,0.4,0.4} \right),\left( {de,0.5,0.4} \right),\left( {ef,0.6,0.3} \right)} \\ \end{array} } \right\}$$

The application of the Definition (2) on the two ${\mathbb{IFS}}$ ${\mathcal{A}}$ and ${\mathcal{B}}$ corresponding to the value $t = 0.70$ gives that:

$${\mathcal{A}}_{0.70} = \left\{ {\left( {a,0.7,0.3} \right),\left( {b,0.7,0.3} \right),\left( {c,0.5,0.4} \right),\left( {d,0.7,0.3} \right),\left( {e,0.6,0.4} \right),\left( {f,0.7,0.3} \right)} \right\}$$

and

$${\mathcal{B}}_{0.70} = \left\{ {\begin{array}{*{20}c} {\left( {ab,0.3,0.3} \right),\left( {ac,0.5,0.4} \right),\left( {af,0.6,0.3} \right),\left( {bc,0.4,0.3} \right),\left( {cd,0.5,0.3} \right),} \\ {\left( {ce,0.4,0.4} \right),\left( {de,0.5,0.4} \right),\left( {ef,0.6,0.3} \right)} \\ \end{array} } \right\}$$

**Figure 1:**
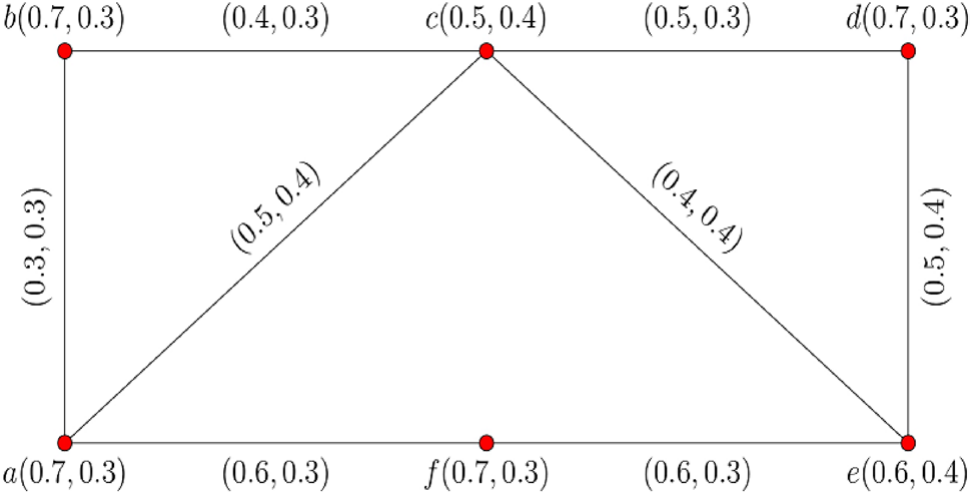
$$0.70 - {\mathbb{IFG}} \,{\mathcal{G}}_{0.7}$$.

The graphical representation of $0.70$- ${\mathbb{IFG}}$ ${\mathcal{G}}_{0.7} = \left\langle {{\mathcal{A}}_{0.7} ,{\mathcal{B}}_{0.7} } \right\rangle$ is displayed in Fig. 1.

### Definition 7

A t-${\mathbb{IFG}}$ ${\mathcal{H}}_{t} = \left\langle {{\mathcal{A}}_{t}^{\prime} ,{\mathcal{B}}_{t}^{\prime} } \right\rangle$ is said to be a t-intuitionistic fuzzy subgraph of t-${\mathbb{IFG}}$ ${\mathcal{G}}_{t} = \left\langle {{\mathcal{A}}_{t} ,{\mathcal{B}}_{t} } \right\rangle$ if ${\mathcal{A}}_{t}^{\prime} \subseteq {\mathcal{A}}_{t}$ and ${\mathcal{B}}_{t}^{\prime} \subseteq {\mathcal{B}}_{t} .$

### Definition 8

A t-${\mathbb{IFG}}$ ${\mathcal{G}}_{t} = \left\langle {{\mathcal{A}}_{t} ,{\mathcal{B}}_{t} } \right\rangle$ is said to be complete t-${\mathbb{IFG}}$ if it admits the following conditions:

$$\begin{aligned} & \mu_{{{\mathcal{B}}_{t} }} \left( {u_{1} ,u_{2} } \right) = min\left\{ {\mu_{{{\mathcal{A}}_{t} }} \left( {u_{1} } \right),\mu_{{{\mathcal{A}}_{t} }} \left( {u_{2} } \right)} \right\} \\ & \sigma_{{{\mathcal{B}}_{t} }} \left( {u_{1} ,u_{2} } \right) = max\left\{ {\sigma_{{{\mathcal{A}}_{t} }} \left( {u_{1} } \right),\sigma_{{{\mathcal{A}}_{t} }} \left( {u_{2} } \right)} \right\},\forall (u_{1} ,u_{2} ) \in {\mathbb{E}}. \\ \end{aligned}$$

### Example 2

**Figure 2:**
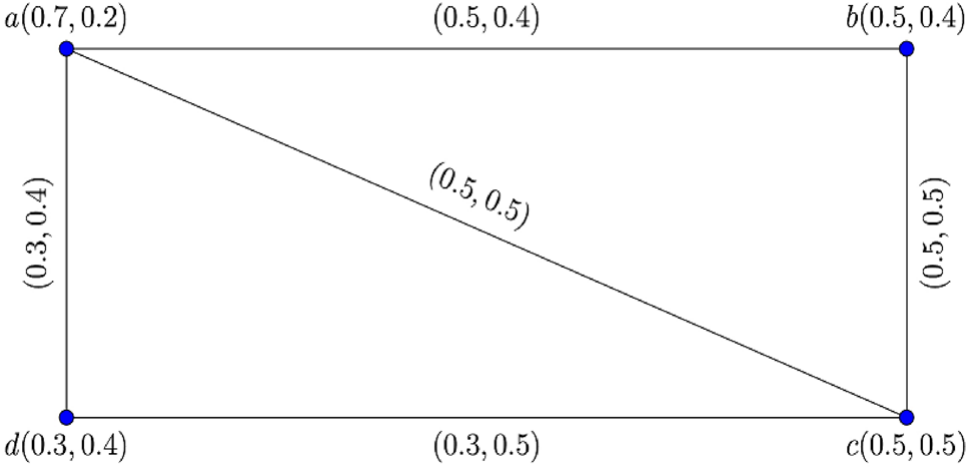
Complete $$0.80 - {\mathbb{IFG}} \,{\mathcal{G}}_{0.80}$$.

Consider the complete $0.80$*-*${\mathbb{IFG}}$ ${\mathcal{G}}_{t}$ as depicted in Fig. 2.

### Definition 9

Let $${\mathcal{G}}_{t} = \left\langle {{\mathcal{A}}_{t} ,{\mathcal{B}}_{t} } \right\rangle$$ be a t-$${\mathbb{IFG}}$$$$,$$ where $${\mathcal{A}}_{t} = \left\langle {\mu_{{{\mathcal{A}}_{t} }} ,\sigma_{{{\mathcal{A}}_{t} }} } \right\rangle$$ is a t-$${\mathbb{IFS}}$$ on $${\mathbb{V}}$$ and $${\mathcal{B}}_{t} = \left\langle {\mu_{{{\mathcal{B}}_{t} }} ,\sigma_{{{\mathcal{B}}_{t} }} } \right\rangle$$ is a t-$${\mathbb{IFS}}$$ on $${\mathbb{E}} \subseteq {\mathbb{V}} \times {\mathbb{V}}.$$ Then.

1. The order of t-${\mathbb{IFG}}$ ${\mathcal{G}}_{t}$ is defined as:
  $${\mathcal{O}}\left( {{\mathcal{G}}_{t} } \right) = \left( {\mathop \sum \limits_{{u_{1} \in {\mathbb{V}}}} \mu_{{{\mathcal{A}}_{t} }} \left( {u_{1} } \right),\mathop \sum \limits_{{u_{1} \in {\mathbb{V}}}} \sigma_{{{\mathcal{A}}_{t} }} \left( {u_{1} } \right)} \right)$$
2. The size of t-${\mathbb{IFG}}$ ${\mathcal{G}}_{t}$ is defined as:
  $${\mathcal{S}}\left( {{\mathcal{G}}_{t} } \right) = \left( {\mathop \sum \limits_{{\left( {u_{1} ,u_{2} } \right) \in {\mathbb{E}}}} \mu_{{{\mathcal{B}}_{t} }} \left( {u_{1} ,u_{2} } \right),\mathop \sum \limits_{{\left( {u_{1} ,u_{2} } \right) \in {\mathbb{E}}}} \sigma_{{{\mathcal{B}}_{t} }} \left( {u_{1} ,u_{2} } \right)} \right)$$

### Example 3

The order of t-${\mathbb{IFG}}$ ${\mathcal{G}}_{t}$ is $\left( {3.9,2} \right).$ (see Example 1).

### Proposition 1

Let $${\mathcal{G}}_{t} = \left\langle {{\mathcal{A}}_{t} ,{\mathcal{B}}_{t} } \right\rangle$$ be a t-$${\mathbb{IFG}}$$$$,$$ then $${\mathcal{S}}\left( {{\mathcal{G}}_{t} } \right) \le {\mathcal{O}}\left( {{\mathcal{G}}_{t} } \right).$$

### Definition 10

Let ${\mathcal{G}}_{t} = \left\langle {{\mathcal{A}}_{t} ,{\mathcal{B}}_{t} } \right\rangle$ be a t-${\mathbb{IFG}}$ on ${\mathbb{G}} = \left\langle {{\mathbb{V}},{\mathbb{E}}} \right\rangle ,$ then:

1. In t-${\mathbb{IFG}}\,\,{\mathcal{G}}_{t}$, the degree of a vertex $u_{1}$ in ${\mathcal{G}}_{t}$ is defined as follows:
  $$deg_{{{\mathcal{G}}_{t} }} \left( {u_{1} } \right) = \left( {deg_{{\mu_{{{\mathcal{B}}_{t} }} }} \left( {u_{1} } \right),deg_{{\sigma_{{{\mathcal{B}}_{t} }} }} \left( {u_{1} } \right)} \right) = \left( {\mathop \sum \limits_{{\left( {u_{1} ,u_{2} } \right) \in {\mathbb{E}}}} \mu_{{{\mathcal{B}}_{t} }} \left( {u_{1} ,u_{2} } \right),\mathop \sum \limits_{{\left( {u_{1} ,u_{2} } \right) \in {\mathbb{E}}}} \sigma_{{{\mathcal{B}}_{t} }} \left( {u_{1} ,u_{2} } \right)} \right)$$
2. The minimum degree $\delta \left( {{\mathcal{G}}_{t} } \right)$ of t-${\mathbb{IFG}}$ ${\mathcal{G}}_{t}$ is given by:
  $$\delta \left( {{\mathcal{G}}_{t} } \right) = \left( {\delta_{{\mu_{{{\mathcal{B}}_{t} }} }} \left( {{\mathcal{G}}_{t} } \right),\delta_{{\sigma_{{{\mathcal{B}}_{t} }} }} \left( {{\mathcal{G}}_{t} } \right)} \right) = \left( {Min\left\{ {deg_{{\mu_{{{\mathcal{B}}_{t} }} }} \left( {u_{1} } \right):u_{1} \in {\mathbb{V}}} \right\},Min\left\{ {deg_{{\sigma_{{{\mathcal{B}}_{t} }} }} \left( {u_{1} } \right):u_{1} \in {\mathbb{V}}} \right\}} \right)$$
3. The maximum degree $\Delta \left( {{\mathcal{G}}_{t} } \right)$ of ${\mathcal{G}}_{t}$ is defined as follows:
  $$\Delta \left( {{\mathcal{G}}_{t} } \right) = \left( {\Delta_{{\mu_{{{\mathcal{B}}_{t} }} }} \left( {{\mathcal{G}}_{t} } \right),\Delta_{{\sigma_{{{\mathcal{B}}_{t} }} }} \left( {{\mathcal{G}}_{t} } \right)} \right) = \left( {Max\left\{ {deg_{{\mu_{{{\mathcal{B}}_{t} }} }} \left( {u_{1} } \right):u_{1} \in {\mathbb{V}}} \right\},Max\left\{ {deg_{{\sigma_{{{\mathcal{B}}_{t} }} }} \left( {u_{1} } \right):u_{1} \in {\mathbb{V}}} \right\}} \right)$$

### Proposition 2.

In t-${\mathbb{IFG}}$ ${ \mathcal{G}}_{t} ,$ then the following inequality holds:

$$\delta \left( {{\mathcal{G}}_{t} } \right) \le \Delta \left( {{\mathcal{G}}_{t} } \right) \le {\mathcal{S}}\left( {{\mathcal{G}}_{t} } \right) \le {\mathcal{O}}\left( {{\mathcal{G}}_{t} } \right).$$

### Example 4.

From Example 1*,* the degree of each vertex in ${\mathcal{G}}_{t}$ are:

$$deg_{{{\mathcal{G}}_{t} }} \left( a \right) = \left( {1.4,1} \right)$$, $$deg_{{{\mathcal{G}}_{t} }} \left( b \right) = \left( {0.7,0.6} \right)$$, $$deg_{{{\mathcal{G}}_{t} }} \left( c \right) = \left( {1,8.1.4} \right)$$, $$deg_{{{\mathcal{G}}_{t} }} \left( d \right) = \left( {1,0.7} \right)$$, $$deg_{{{\mathcal{G}}_{t} }} \left( e \right) = \left( {1.5,1.1} \right)$$*,* and $$deg_{{{\mathcal{G}}_{t} }} \left( f \right) = \left( {1.2,0.6} \right)$$*.*

### Theorem 1.

Let ${\mathcal{G}}_{t} = \left\langle {{\mathcal{A}}_{t} ,{\mathcal{B}}_{t} } \right\rangle$ be any t-${\mathbb{IFG}}$, then:

$$\sum deg_{{{\mathcal{G}}_{t} }} \left( {u_{i} } \right) = \left( {2\sum \mu_{{{\mathcal{B}}_{t} }} \left( {u_{i} ,w} \right),2\sum \sigma_{{{\mathcal{B}}_{t} }} \left( {u_{i} ,w} \right)} \right)$$

### Proof.

Let $${\mathcal{G}}_{t} = \left\langle {{\mathcal{A}}_{t} ,{\mathcal{B}}_{t} } \right\rangle$$ be a t-$${\mathbb{IFG}}$$*.*

Consider

$$\sum deg_{{{\mathcal{G}}_{t} }} \left( {u_{i} } \right) = \left( {\sum deg_{{\mu_{{{\mathcal{B}}_{t} }} }} \left( {u_{i} } \right),\sum deg_{{\sigma_{{{\mathcal{B}}_{t} }} }} \left( {u_{i} } \right)} \right)$$

The application of the part (1) of Definition (9) to gives that:

$$\begin{aligned} & = \left( {deg_{{\mu_{{{\mathcal{B}}_{t} }} }} \left( {u_{1} } \right),deg_{{\mu_{{{\mathcal{B}}_{t} }} }} \left( {u_{1} } \right)} \right) + \left( {deg_{{\mu_{{{\mathcal{B}}_{t} }} }} \left( {u_{2} } \right),deg_{{\mu_{{{\mathcal{B}}_{t} }} }} \left( {u_{2} } \right)} \right) + \cdots + \left( {deg_{{\mu_{{{\mathcal{B}}_{t} }} }} \left( {u_{n} } \right),deg_{{\mu_{{{\mathcal{B}}_{t} }} }} \left( {u_{n} } \right)} \right) \\ & \quad = \left( {\mu_{{{\mathcal{B}}_{t} }} \left( {u_{1} ,u_{2} } \right),\sigma_{{{\mathcal{B}}_{t} }} \left( {u_{1} ,u_{2} } \right)} \right) + \left( {\mu_{{{\mathcal{B}}_{t} }} \left( {u_{1} ,u_{3} } \right),\sigma_{{{\mathcal{B}}_{t} }} \left( {u_{1} ,u_{3} } \right)} \right) + \cdots + \left( {\mu_{{{\mathcal{B}}_{t} }} \left( {u_{1} ,u_{n} } \right),\sigma_{{{\mathcal{B}}_{t} }} \left( {u_{1} ,u_{n} } \right)} \right) \\ & \quad \quad + \left( {\mu_{{{\mathcal{B}}_{t} }} \left( {u_{2} ,u_{1} } \right),\sigma_{{{\mathcal{B}}_{t} }} \left( {u_{2} ,u_{1} } \right)} \right) + \left( {\mu_{{{\mathcal{B}}_{t} }} \left( {u_{2} ,u_{3} } \right),\sigma_{{{\mathcal{B}}_{t} }} \left( {u_{2} ,u_{3} } \right)} \right) + \cdots + \left( {\mu_{{{\mathcal{B}}_{t} }} \left( {u_{2} ,u_{n} } \right),\sigma_{{{\mathcal{B}}_{t} }} \left( {u_{2} ,u_{n} } \right)} \right) \\ & \quad \quad + \ldots + \left( {\mu_{{{\mathcal{B}}_{t} }} \left( {u_{n} ,u_{1} } \right),\sigma_{{{\mathcal{B}}_{t} }} \left( {u_{n} ,u_{1} } \right)} \right) + \left( {\mu_{{{\mathcal{B}}_{t} }} \left( {u_{n} ,u_{2} } \right),\sigma_{{{\mathcal{B}}_{t} }} \left( {u_{n} ,u_{2} } \right)} \right) + \cdots + \left( {\mu_{{{\mathcal{B}}_{t} }} \left( {u_{n - 1} ,u_{n} } \right),\sigma_{{{\mathcal{B}}_{t} }} \left( {u_{n - 1} ,u_{n} } \right)} \right) \\ & \quad = 2\left( {\mu_{{{\mathcal{B}}_{t} }} \left( {u_{1} ,u_{2} } \right),\sigma_{{{\mathcal{B}}_{t} }} \left( {u_{1} ,u_{2} } \right)} \right) + 2\left( {\mu_{{{\mathcal{B}}_{t} }} \left( {u_{1} ,u_{3} } \right),\sigma_{{{\mathcal{B}}_{t} }} \left( {u_{1} ,u_{3} } \right)} \right) + \cdots + 2\left( {\mu_{{{\mathcal{B}}_{t} }} \left( {u_{1} ,u_{n} } \right),\sigma_{{{\mathcal{B}}_{t} }} \left( {u_{1} ,u_{n} } \right)} \right) \\ & \quad = \left( {2\sum \mu_{{{\mathcal{B}}_{t} }} \left( {u_{i} ,{ }w} \right),2\sum \sigma_{{{\mathcal{B}}_{t} }} \left( {u_{i} ,w} \right)} \right) \\ \end{aligned}$$

Hence, it completes the proof.

### Corollary 1.

In a t-${\mathbb{IFG}}$, the odd membership degree and the odd non-membership degree have an even number of vertices.

### Corollary 2.

In a t-${\mathbb{IFG}}$*,* $n - 1$ is the maximum degree of any vertex $n$*.*

## Operations on t-intuitionistic fuzzy graph

This section explores the set-theoretical operations of t-$${\mathbb{IFG}}$$$$.$$ We also establish and analyze the fundamental characteristics of these phenomena.

### Definition 11.

Let ${\mathcal{G}}_{1}^{t} = \left\langle {{\mathcal{A}}_{t} ,{\mathcal{B}}_{t} } \right\rangle$ and ${\mathcal{G}}_{2}^{t} = \left\langle {{\mathcal{A}}_{t}^{\prime} ,{\mathcal{B}}_{t}^{\prime} } \right\rangle$ be any two t-${\mathbb{IFG}}$ of ${\mathbb{G}}_{1} = \left\langle {{\mathbb{V}},{\mathbb{E}}} \right\rangle$ and ${\mathbb{G}}_{2} = \left\langle {{\mathbb{V}}^{\prime}},{{\mathbb{E}}^{\prime}} \right\rangle ,$ respectively. The Cartesian product ${\mathcal{G}}_{1}^{t} \times {\mathcal{G}}_{2}^{t}$ of t-${\mathbb{IFG}}$ ${\mathcal{G}}_{1}^{t}$ and ${\mathcal{G}}_{2}^{t}$ is defined by $\left\langle {{\mathcal{A}}_{t} \times {\mathcal{A}}_{t}^{\prime} ,{\mathcal{B}}_{t} \times {\mathcal{B}}_{t}^{\prime} } \right\rangle$, where ${\mathcal{A}}_{t} \times {\mathcal{A}}_{t}^{\prime}$ and ${\mathcal{B}}_{t} \times {\mathcal{B}}_{t}^{\prime}$ are t-${\mathbb{IFS}}$ on ${\mathbb{V}} \times {{\mathbb{V}}^{\prime}} = \{ \left( {u_{1} ,w_{1} ),(u_{2} ,w_{2} } \right):u_{1} ,u_{2} \in {\mathbb{V}} \wedge w_{1} ,w_{2} \in {{\mathbb{V}}^{\prime}}$} and ${\mathbb{E}} \times {{\mathbb{E}}^{\prime}} = \left\{ {\left( {u_{1} ,w_{1} ),(u_{2} ,w_{2} } \right):u_{1} = u_{2} ,u_{1} ,u_{2} \in {\mathbb{V}},\left( {w_{1} ,w_{2} } \right) \in {{\mathbb{E}}^{\prime}}} \right\} \cup \left\{ {\left( {u_{1} ,w_{1} ),(u_{2} ,w_{2} } \right):w_{1} = w_{2} ,w_{1} ,w_{2} \in {{\mathbb{V}}^{\prime}} ,\left( {u_{1} ,u_{2} } \right) \in {\mathbb{E}}} \right\} \cup \left\{ {\left( {u_{1} ,w_{1} ),(u_{2} ,w_{2} } \right):w_{1} \ne w_{2} ,w_{1} ,w_{2} \in {{\mathbb{V}}^{\prime}} ,\left( {u_{1} ,u_{2} } \right) \in {\mathbb{E}}} \right\},$ respectively, which satisfies the following conditions:

1. $$\forall \left( {u_{1} ,w_{1} } \right) \in {\mathbb{V}} \times {{\mathbb{V}}^{\prime}}$$
  - $$\mu_{{{\mathcal{A}}_{t} \times {\mathcal{A}}_{t}^{\prime} }} \left( {u_{1} ,w_{1} } \right) = min\left\{ {\mu_{{{\mathcal{A}}_{t} }} \left( {u_{1} } \right),\mu_{{{\mathcal{A}}_{t}^{\prime} }} \left( {w_{1} } \right)} \right\}$$
  - $$\sigma_{{{\mathcal{A}}_{t} \times {\mathcal{A}}_{t}^{\prime} }} \left( {u_{1} ,w_{1} } \right) = max\left\{ {\sigma_{{{\mathcal{A}}_{t} }} \left( {u_{1} } \right),\sigma_{{{\mathcal{A}}_{t}^{\prime} }} \left( {w_{1} } \right)} \right\}$$
2. If $$u_{1} = u_{2}$$ and $$\forall \left( {w_{1} ,w_{2} } \right) \in {{\mathbb{E}}^{\prime}}$$
  - $$\mu_{{{\mathcal{B}}_{t} \times {\mathcal{B}}_{t}^{\prime} }} \left( {\left( {u_{1} ,w_{1} ),(u_{2} ,w_{2} } \right)} \right) = min\left\{ {\mu_{{{\mathcal{A}}_{t} }} \left( {u_{1} } \right),\mu_{{{\mathcal{B}}_{t}^{\prime} }} \left( {w_{1} ,w_{2} } \right)} \right\}$$
  - $$\sigma_{{{\mathcal{B}}_{t} \times {\mathcal{B}}_{t}^{\prime} }} \left( {\left( {u_{1} ,w_{1} ),(u_{2} ,w_{2} } \right)} \right) = max\left\{ {\sigma_{{{\mathcal{A}}_{t} }} \left( {u_{1} } \right),\sigma_{{{\mathcal{B}}_{t}^{\prime} }} \left( {w_{1} ,w_{2} } \right)} \right\}$$
3. If $$w_{1} = w_{2}$$ and $$\forall \left( {u_{1} ,u_{2} } \right) \in {\mathbb{E}}$$
  - $$\mu_{{{\mathcal{B}}_{t} \times {\mathcal{B}}_{t}^{\prime} }} \left( {\left( {u_{1} ,w_{1} ),(u_{2} ,w_{2} } \right)} \right) = min\left\{ {\mu_{{{\mathcal{B}}_{t} }} \left( {u_{1} ,u_{2} } \right),\mu_{{{\mathcal{A}}_{t}^{\prime} }} \left( {w_{1} } \right)} \right\}$$
  - $$\sigma_{{{\mathcal{B}}_{t} \times {\mathcal{B}}_{t}^{\prime} }} \left( {\left( {u_{1} ,w_{1} ),(u_{2} ,w_{2} } \right)} \right) = max\left\{ {\sigma_{{{\mathcal{B}}_{t} }} \left( {u_{1} ,u_{2} } \right),\sigma_{{{\mathcal{A}}_{t}^{\prime} }} \left( {w_{1} } \right)} \right\}$$

### Example 5.

**Figure 3:**
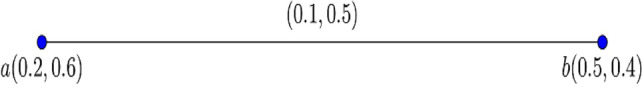
$$0.8 - {\mathbb{IFG}} \,{\mathcal{G}}_{1}^{0.8}$$.

**Figure 4:**
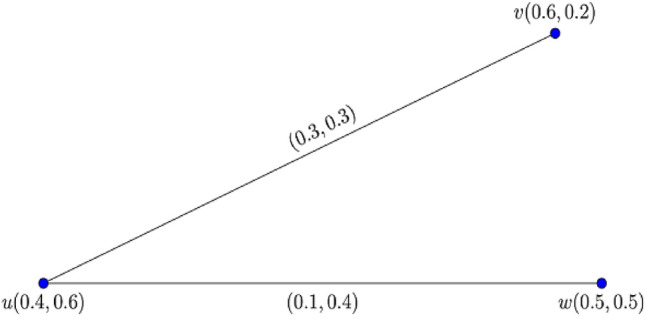
$$0.8 - {\mathbb{IFG}} \,{\mathcal{G}}_{2}^{0.8}$$.

Consider the two $0.8$-${\mathbb{IFG}}$ ${\mathcal{G}}_{1}^{t}$ and ${\mathcal{G}}_{2}^{t}$ illustrated in Figs. 3 and 4.

**Figure 5:**
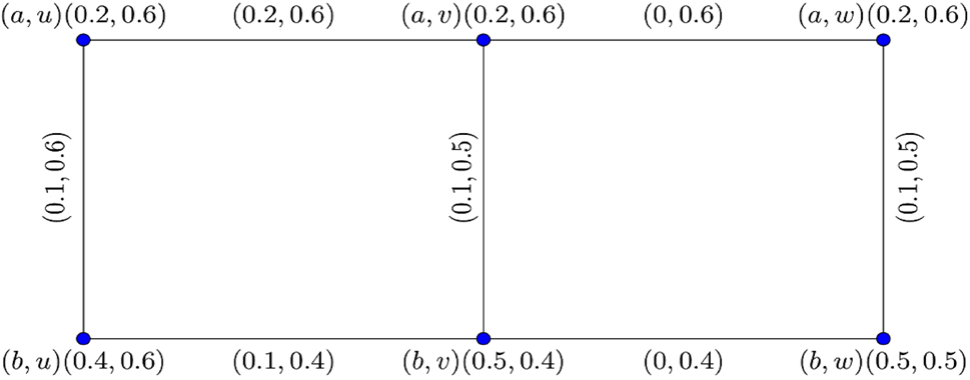
$$0.8 -$$$${\mathbb{IFG}}$$ of $${\mathcal{G}}_{1}^{0.8} \times {\mathcal{G}}_{2}^{0.8}$$.

Figure 5 shows their corresponding Cartesian product ${\mathcal{G}}_{1}^{0.8} \times {\mathcal{G}}_{2}^{0.8} :$

### Definition 12.

The degree of a vertex in ${\mathcal{G}}_{1}^{t} \times {\mathcal{G}}_{2}^{t}$ is defined as follows: for any $\left( {u_{1} ,w_{1} } \right) \in {\mathbb{V}} \times {{\mathbb{V}}^{\prime}}$.

$$deg_{{{\mathcal{G}}_{1} \times {\mathcal{G}}_{2} }} \left( {u_{1} ,w_{1} } \right) = \left( {deg\left( {\mu_{{{\mathcal{B}}_{t} \times {\mathcal{B}}_{t}^{\prime} }} \left( {\left( {u_{1} ,w_{1} ),(u_{2} ,w_{2} } \right)} \right)} \right),deg\left( {\sigma_{{{\mathcal{B}}_{t} \times {\mathcal{B}}_{t}^{\prime} }} \left( {\left( {u_{1} ,w_{1} ),(u_{2} ,w_{2} } \right)} \right)} \right)} \right)$$

where

$$\begin{aligned} deg\left( {\mu_{{{\mathcal{B}}_{t} \times {\mathcal{B}}_{t}^{\prime} }} \left( {\left( {u_{1} ,w_{1} ),(u_{2} ,w_{2} } \right)} \right)} \right) = & \mathop \sum \limits_{{u_{1} = u_{2} ,\left( {w_{1} ,w_{2} } \right) \in {{\mathbb{E}}^{\prime}}}} min\left\{ {\mu_{{{\mathcal{A}}_{t} }} \left( {u_{1} } \right),\mu_{{{\mathcal{B}}_{t}^{\prime} }} \left( {w_{1} ,w_{2} } \right)} \right\} \\ & + \mathop \sum \limits_{{w_{1} = w_{2} ,\left( {u_{1} ,u_{2} } \right) \in {\mathbb{E}}}} min\left\{ {\mu_{{{\mathcal{B}}_{t} }} \left( {u_{1} ,u_{2} } \right),\mu_{{{\mathcal{A}}_{t}^{\prime} }} \left( {w_{1} } \right)} \right\} \\ \end{aligned}$$

and

$$\begin{aligned} deg\left( {\sigma_{{{\mathcal{B}}_{t} \times {\mathcal{B}}_{t}^{\prime} }} \left( {\left( {u_{1} ,w_{1} ),(u_{2} ,w_{2} } \right)} \right)} \right) = & \mathop \sum \limits_{{u_{1} = u_{2} ,\left( {w_{1} ,v_{2} } \right) \in {{\mathbb{E}}^{\prime}}}} max\left\{ {\sigma_{{{\mathcal{A}}_{t} }} \left( {u_{1} } \right),\sigma_{{{\mathcal{B}}_{t}^{\prime} }} \left( {w_{1} ,w_{2} } \right)} \right\} \\ & + \mathop \sum \limits_{{w_{1} = w_{2} ,\left( {u_{1} ,u_{2} } \right) \in {\mathbb{E}}}} max\left\{ {\sigma_{{{\mathcal{B}}_{t} }} \left( {u_{1} ,u_{2} } \right),\sigma_{{{\mathcal{A}}_{t}^{\prime} }} \left( {w_{1} } \right)} \right\} \\ \end{aligned}$$

### Example 6.

According to Example 5*,* each vertex in ${\mathcal{G}}_{1}^{t} \times {\mathcal{G}}_{2}^{t} \user2{ }$ has the following degree:

$$\begin{aligned} & deg_{{{\mathcal{G}}_{1}^{t} \times {\mathcal{G}}_{2}^{t} }} \left( {a,u} \right) = \left( {0.3,1.3} \right),\;deg_{{{\mathcal{G}}_{1}^{t} \times {\mathcal{G}}_{2}^{t} }} \left( {a,v} \right) = \left( {0.5,1.9} \right),\;deg_{{{\mathcal{G}}_{1}^{t} \times {\mathcal{G}}_{2}^{t} }} \left( {a,w} \right) = \left( {0.3,1.3} \right) \\ & deg_{{{\mathcal{G}}_{1}^{t} \times {\mathcal{G}}_{2}^{t} }} \left( {b,u} \right) = \left( {0.6,1.2} \right),\;deg_{{{\mathcal{G}}_{1}^{t} \times {\mathcal{G}}_{2}^{t} }} \left( {b,v} \right) = \left( {0.6,1.6} \right),\;{\text{and}}\;deg_{{{\mathcal{G}}_{1}^{t} \times {\mathcal{G}}_{2}^{t} }} \left( {b,w} \right) = \left( {0.1,1.1} \right). \\ \end{aligned}$$

### Proposition 3.

The Cartesian product of two t-$${\mathbb{IFG}}$$ is a t-$${\mathbb{IFG}}$$$$.$$

### Proof.

The condition for ${\mathcal{A}}_{t} \times {\mathcal{A}}_{t}^{\prime}$ is self-explanatory. Let $u_{1} \in {\mathbb{V}}$ and $\left( {w_{1} ,w_{2} } \right) \in {{\mathbb{E}}^{\prime}}.$ Then:

$$\begin{aligned} \mu_{{{\mathcal{B}}_{t} \circ {\mathcal{B}}_{t}^{\prime} }} \left( {\left( {u_{1} ,w_{1} ),(u_{1} ,w_{2} } \right)} \right) = & \,min\left\{ {\mu_{{{\mathcal{A}}_{t} }} \left( {u_{1} } \right),\mu_{{{\mathcal{B}}_{t}^{\prime} }} \left( {w_{1} ,w_{2} } \right)} \right\} \\ \le &\, min\left\{ {\mu_{{{\mathcal{A}}_{t} }} \left( {u_{1} } \right),min\left\{ {\mu_{{{\mathcal{A}}_{t}^{\prime} }} \left( {w_{1} } \right),\mu_{{{\mathcal{A}}_{t}^{\prime} }} \left( {w_{2} } \right)} \right\}} \right\} \\ \le &\, min\left\{ {min\left\{ {\mu_{{{\mathcal{A}}_{t} }} \left( {u_{1} } \right),\mu_{{{\mathcal{A}}_{t}^{\prime} }} \left( {w_{1} } \right)} \right\},min\left\{ {\mu_{{{\mathcal{A}}_{t} }} \left( {u_{1} } \right),\mu_{{{\mathcal{A}}_{t}^{\prime} }} \left( {w_{2} } \right)} \right\}} \right\} \\ = &\, min\left\{ {\mu_{{{\mathcal{A}}_{t} \times {\mathcal{A}}_{t}^{\prime} }} \left( {u_{1} ,w_{1} } \right),\mu_{{{\mathcal{A}}_{t} \times {\mathcal{A}}_{t}^{\prime} }} \left( {u_{1} ,w_{2} } \right)} \right\} \\ \end{aligned}$$

Consequently $$\mu_{{{\mathcal{B}}_{t} \circ {\mathcal{B}}_{t}^{\prime} }} \left( {\left( {u_{1} ,w_{1} ),(u_{1} ,w_{2} } \right)} \right) \le min\left\{ {\mu_{{{\mathcal{A}}_{t} \times {\mathcal{A}}_{t}^{\prime} }} \left( {u_{1} ,w_{1} } \right),\mu_{{{\mathcal{A}}_{t} \times {\mathcal{A}}_{t}^{\prime} }} \left( {u_{1} ,w_{2} } \right)} \right\}, {\text{if}} \;u_{1} \in {\mathbb{V}}$$ and $$\left( {w_{1} ,w_{2} } \right) \in {{\mathbb{E}}^{\prime}}.$$

Also

$$\begin{aligned} \sigma_{{{\mathcal{B}}_{t} \circ {\mathcal{B}}_{t}^{\prime} }} \left( {\left( {u_{1} ,w_{1} ),(u_{1} ,w_{2} } \right)} \right) = &\, max\left\{ {\sigma_{{{\mathcal{A}}_{t} }} \left( {u_{1} } \right),\sigma_{{{\mathcal{B}}_{t}^{\prime} }} \left( {w_{1} ,w_{2} } \right)} \right\} \\ \le &\, max\left\{ {\sigma_{{{\mathcal{A}}_{t} }} \left( {u_{1} } \right),max\left\{ {\sigma_{{{\mathcal{A}}_{t}^{\prime} }} \left( {w_{1} } \right),\sigma_{{{\mathcal{A}}_{t}^{\prime} }} \left( {w_{2} } \right)} \right\}} \right\} \\ \le & \, max\left\{ {max\left\{ {\sigma_{{{\mathcal{A}}_{t} }} \left( {u_{1} } \right),\sigma_{{{\mathcal{A}}_{t}^{\prime} }} \left( {w_{1} } \right)} \right\},max\left\{ {\sigma_{{{\mathcal{A}}_{t} }} \left( {u_{1} } \right),\sigma_{{{\mathcal{A}}_{t}^{\prime} }} \left( {w_{2} } \right)} \right\}} \right\} \\ = &\, max\left\{ {\sigma_{{{\mathcal{A}}_{t} \times {\mathcal{A}}_{t}^{\prime} }} \left( {u_{1} ,w_{1} } \right),\sigma_{{{\mathcal{A}}_{t} \times {\mathcal{A}}_{t}^{\prime} }} \left( {u_{1} ,w_{2} } \right)} \right\} \\ \end{aligned}$$

Thus $$\sigma_{{{\mathcal{B}}_{t} \circ {\mathcal{B}}_{t}^{\prime} }} \left( {\left( {u_{1} ,w_{1} ),(u_{1} ,w_{2} } \right)} \right) \le max\left\{ {\sigma_{{{\mathcal{A}}_{t} \times {\mathcal{A}}_{t}^{\prime} }} \left( {u_{1} ,w_{1} } \right),\sigma_{{{\mathcal{A}}_{t} \times {\mathcal{A}}_{t}^{\prime} }} \left( {u_{1} ,w_{2} } \right)} \right\},{\text{if}}\;u{\ominus }_{1} \in {\mathbb{V}}$$ and $$\left( {w_{1} ,w_{2} } \right) \in {{\mathbb{E}}^{\prime}}.$$

Likewise, we can demonstrate it for $w_{1} \in {{\mathbb{V}}^{\prime}}$ and $\left( {u_{1} ,u_{2} } \right) \in {\mathbb{E}}$*.*

### Definition 13.

The composition ${\mathcal{G}}_{1}^{t} \circ {\mathcal{G}}_{2}^{t}$ of two t-${\mathbb{IFG}}$ ${\mathcal{G}}_{1}^{t}$ and ${\mathcal{G}}_{2}^{t}$ is a t-${\mathbb{IFG}}$ and defined as a pair $\left\langle {{\mathcal{A}}_{t} \circ {\mathcal{A}}_{t}^{\prime} ,{\mathcal{B}}_{t} \circ {\mathcal{B}}_{t}^{\prime} } \right\rangle$, where ${\mathcal{A}}_{t} \circ {\mathcal{A}}_{t}^{\prime}$ and ${\mathcal{B}}_{t} \circ {\mathcal{B}}_{t}^{\prime}$ are t-${\mathbb{IFS}}$ on ${\mathbb{V}} \times {{\mathbb{V}}^{\prime}} = \{ \left( {u_{1} ,w_{1} ),(u_{2} ,w_{2} } \right):u_{1} ,u_{2} \in {\mathbb{V}} \wedge w_{1} ,w_{2} \in {{\mathbb{V}}^{\prime}}$} and ${\mathbb{E}} \times {{\mathbb{E}}^{\prime}} = \left\{ {\left( {u_{1} ,w_{1} ),(u_{2} ,w_{2} } \right):u_{1} = u_{2} ,u_{1} ,u_{2} \in {\mathbb{V}},\left( {w_{1} ,w_{2} } \right) \in {{\mathbb{E}}^{\prime}}} \right\} \cup \left\{ {\left( {u_{1} ,w_{1} ),(u_{2} ,w_{2} } \right):w_{1} = w_{2} ,w_{1} ,w_{2} \in {{\mathbb{V}}^{\prime}} ,\left( {u_{1} ,u_{2} } \right) \in {\mathbb{E}}} \right\} \cup \left\{ {\left( {u_{1} ,w_{1} ),(u_{2} ,w_{2} } \right):w_{1} \ne w_{2} ,w_{1} ,w_{2} \in {{\mathbb{V}}^{\prime}} ,\left( {u_{1} ,u_{2} } \right) \in {\mathbb{E}}} \right\},$ respectively, which satisfies the following conditions:

1. $$\forall \left( {u_{1} ,w_{1} } \right) \in {\mathbb{V}} \times {{\mathbb{V}}^{\prime}}$$
  - $$\mu_{{{\mathcal{A}}_{t} \circ {\mathcal{A}}_{t}^{\prime} }} \left( {u_{1} ,w_{1} } \right) = min\left\{ {\mu_{{{\mathcal{A}}_{t} }} \left( {u_{1} } \right),\mu_{{{\mathcal{A}}_{t}^{\prime} }} \left( {w_{1} } \right)} \right\}$$
  - $$\sigma_{{{\mathcal{A}}_{t} \circ {\mathcal{A}}_{t}^{\prime} }} \left( {u_{1} ,w_{1} } \right) = max\left\{ {\sigma_{{{\mathcal{A}}_{t} }} \left( {u_{1} } \right),\sigma_{{{\mathcal{A}}_{t}^{\prime} }} \left( {w_{1} } \right)} \right\}$$
2. If $$u_{1} = u_{2}$$ and $$\forall (w_{1} ,w_{2} ) \in {{\mathbb{E}}^{\prime}}$$
  - $$\mu_{{{\mathcal{B}}_{t} \circ {\mathcal{B}}_{t}^{\prime} }} \left( {\left( {u_{1} ,w_{1} ),(u_{2} ,w_{2} } \right)} \right) = min\left\{ {\mu_{{{\mathcal{A}}_{t} }} \left( {u_{1} } \right),\mu_{{{\mathcal{B}}_{t}^{\prime} }} \left( {w_{1} ,w_{2} } \right)} \right\}$$
  - $$\sigma_{{{\mathcal{B}}_{t} \circ {\mathcal{B}}_{t}^{\prime} }} \left( {\left( {u_{1} ,w_{1} ),(u_{2} ,w_{2} } \right)} \right) = max\left\{ {\sigma_{{{\mathcal{A}}_{t} }} \left( {u_{1} } \right),\sigma_{{{\mathcal{B}}_{t}^{\prime} }} \left( {w_{1} ,w_{2} } \right)} \right\}$$
3. If $$w_{1} = w_{2}$$ and $$\forall (u_{1} ,u_{2} ) \in {\mathbb{E}}$$
  - $$\mu_{{{\mathcal{B}}_{t} \circ {\mathcal{B}}_{t}^{\prime} }} \left( {\left( {u_{1} ,w_{1} ),(u_{2} ,w_{2} } \right)} \right) = min\left\{ {\mu_{{{\mathcal{B}}_{t} }} \left( {u_{1} ,u_{2} } \right),\mu_{{{\mathcal{A}}_{t}^{\prime} }} \left( {w_{1} } \right)} \right\}$$
  - $$\sigma_{{{\mathcal{B}}_{t} \circ {\mathcal{B}}_{t}^{\prime} }} \left( {\left( {u_{1} ,w_{1} ),(u_{2} ,w_{2} } \right)} \right) = max\left\{ {\sigma_{{{\mathcal{B}}_{t} }} \left( {u_{1} ,u_{2} } \right),\sigma_{{{\mathcal{A}}_{t}^{\prime} }} \left( {w_{1} } \right)} \right\}$$
4. If $$w_{1} \ne w_{2}$$ and $$\forall (u_{1} ,u_{2} ) \in {\mathbb{E}}$$
  - $$\mu_{{{\mathcal{B}}_{t} \circ {\mathcal{B}}_{t}^{\prime} }} \left( {\left( {u_{1} ,w_{1} ),(u_{2} ,w_{2} } \right)} \right) = min\left\{ {\mu_{{{\mathcal{B}}_{t} }} \left( {u_{1} ,u_{2} } \right),\mu_{{{\mathcal{A}}_{t}^{\prime} }} \left( {w_{1} } \right),\mu_{{{\mathcal{A}}_{t}^{\prime} }} \left( {w_{2} } \right)} \right\}$$
  - $$\sigma_{{{\mathcal{B}}_{t} \circ {\mathcal{B}}_{t}^{\prime} }} \left( {\left( {u_{1} ,w_{1} ),(u_{2} ,w_{2} } \right)} \right) = max\left\{ {\sigma_{{{\mathcal{B}}_{t} }} \left( {u_{1} ,u_{2} } \right),\sigma_{{{\mathcal{A}}_{t}^{\prime} }} \left( {w_{1} } \right),\sigma_{{{\mathcal{A}}_{t}^{\prime} }} \left( {w_{2} } \right)} \right\}$$

### Example 7.

**Figure 6:**
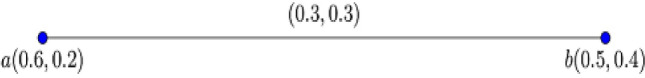
$$0.9 - {\mathbb{IFG}}$$ $${\mathcal{G}}_{1}^{0.9}$$.

**Figure 7:**
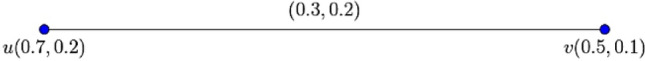
$$0.9 - {\mathbb{IFG}}$$ $${\mathcal{G}}_{2}^{0.9}$$.

Consider the two $0.9$*-*${\mathbb{IFG}}$ ${\mathcal{G}}_{1}^{t}$ and ${\mathcal{G}}_{2}^{t}$ as shown in Figs. 6 and 7.

**Figure 8:**
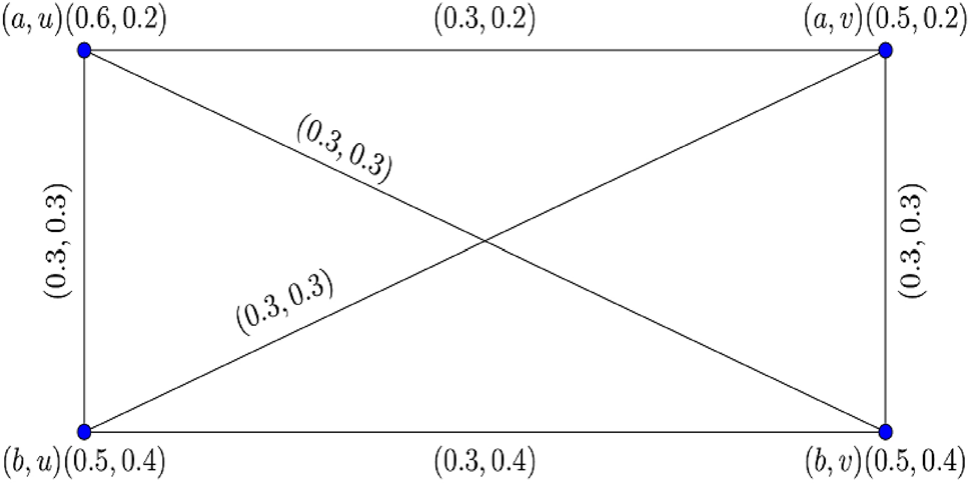
Graphical representation of ${\mathcal{G}}_{1}^{0.9} \circ {\mathcal{G}}_{2}^{0.9}$.

Then, their corresponding composition ${\mathcal{G}}_{1}^{t} \circ {\mathcal{G}}_{2}^{t}$ is shown in Fig. 8.

### Definition 14.

The degree of a vertex in ${\mathcal{G}}_{1}^{t} \circ {\mathcal{G}}_{2}^{t}$ is defined as follows: for any $\left( {u_{1} ,w_{1} } \right) \in {\mathbb{V}} \times {{\mathbb{V}}^{\prime}}$.

$$deg_{{{\mathcal{G}}_{1}^{t} \circ {\mathcal{G}}_{2}^{t} }} \left( {u_{1} ,w_{1} } \right) = \left( {deg\left( {\mu_{{{\mathcal{B}}_{t} \circ {\mathcal{B}}_{t}^{\prime} }} \left( {\left( {u_{1} ,w_{1} ),(u_{2} ,w_{2} } \right)} \right)} \right),deg\left( {\sigma_{{{\mathcal{B}}_{t} \circ {\mathcal{B}}_{t}^{\prime} }} \left( {\left( {u_{1} ,w_{1} ),(u_{2} ,w_{2} } \right)} \right)} \right)} \right)$$

where

$$\begin{aligned} deg\left( {\mu_{{{\mathcal{B}}_{t} \circ {\mathcal{B}}_{t}^{\prime} }} \left( {\left( {u_{1} ,w_{1} ),(u_{2} ,w_{2} } \right)} \right)} \right) = & \mathop \sum \limits_{{u_{1} = u_{2} ,\left( {w_{1} ,w_{2} } \right) \in {{\mathbb{E}}^{\prime}}}} min\left\{ {\mu_{{{\mathcal{A}}_{t} }} \left( {u_{1} } \right),\mu_{{{\mathcal{B}}_{t}^{\prime} }} \left( {w_{1} ,w_{2} } \right)} \right\} \\ & + \mathop \sum \limits_{{w_{1} = w_{2} ,\left( {u_{1} ,u_{2} } \right) \in {\mathbb{E}}}} \min \left\{ {\mu_{{{\mathcal{B}}_{t} }} \left( {u_{1} ,u_{2} } \right),\mu_{{{\mathcal{A}}_{t}^{\prime} }} \left( {w_{1} } \right)} \right\} \\ & + \mathop \sum \limits_{{w_{1} \ne w_{2} ,\left( {u_{1} ,u_{2} } \right) \in {\mathbb{E}}}} min\left\{ {\mu_{{{\mathcal{B}}_{t} }} \left( {u_{1} ,u_{2} } \right),\mu_{{{\mathcal{A}}_{t}^{\prime} }} \left( {w_{1} } \right),\mu_{{{\mathcal{A}}_{t}^{\prime} }} \left( {w_{2} } \right)} \right\} \\ \end{aligned}$$

and

$$\begin{aligned} deg\left( {\sigma_{{{\mathcal{B}}_{t} \circ {\mathcal{B}}_{t}^{\prime} }} \left( {\left( {u_{1} ,w_{1} ),(u_{2} ,w_{2} } \right)} \right)} \right) = & \mathop \sum \limits_{{u_{1} = u_{2} ,\left( {w_{1} ,w_{2} } \right) \in {{\mathbb{E}}^{\prime}}}} max\left\{ {\sigma_{{{\mathcal{A}}_{t} }} \left( {u_{1} } \right),\sigma_{{{\mathcal{B}}_{t}^{\prime} }} \left( {w_{1} ,w_{2} } \right)} \right\} \\ & + \mathop \sum \limits_{{w_{1} = w_{2} ,\left( {u_{1} ,u_{2} } \right) \in {\mathbb{E}}}} \max \left\{ {\sigma_{{{\mathcal{B}}_{t} }} \left( {u_{1} ,u_{2} } \right),\sigma_{{{\mathcal{A}}_{t}^{\prime} }} \left( {w_{1} } \right)} \right\} \\ & + \mathop \sum \limits_{{w_{1} \ne w_{2} ,\left( {u_{1} ,u_{2} } \right) \in {\mathbb{E}}}} max\left\{ {\sigma_{{{\mathcal{B}}_{t} }} \left( {u_{1} ,u_{2} } \right),\sigma_{{{\mathcal{A}}_{t}^{\prime} }} \left( {w_{1} } \right),\sigma_{{{\mathcal{A}}_{t}^{\prime} }} \left( {w_{2} } \right)} \right\}. \\ \end{aligned}$$

### Example 8.

From Example 7, the degree of each vertex in ${\mathcal{G}}_{1}^{t} \circ {\mathcal{G}}_{2}^{t}$ are:

$$deg_{{{\mathcal{G}}_{1}^{t} \circ {\mathcal{G}}_{2}^{t} }} \left( {a,u} \right) = \left( {0.9,1.4} \right)$$*,* $$deg_{{{\mathcal{G}}_{1}^{t} \circ {\mathcal{G}}_{2}^{t} }} \left( {a,v} \right) = \left( {0.9,1.4} \right)$$*,* $$deg_{{{\mathcal{G}}_{1}^{t} \circ {\mathcal{G}}_{2}^{t} }} \left( {b,u} \right) = \left( {0.9,1.6} \right)$$, and $$deg_{{{\mathcal{G}}_{1}^{t} \circ {\mathcal{G}}_{2}^{t} }} \left( {b,v} \right) = \left( {0.9,1.6} \right)$$*.*

### Proposition 4.

Let $${\mathcal{G}}_{1}^{t}$$ and $${\mathcal{G}}_{2}^{t}$$ be any two t-$${\mathbb{IFG}}$$ then $${\mathcal{G}}_{1}^{t} \circ {\mathcal{G}}_{2}^{t}$$ is also a t-$${\mathbb{IFG}}$$$$.$$

### Definition 15.

Let ${\mathcal{G}}_{1}^{t} = \left\langle {{\mathcal{A}}_{t} ,{\mathcal{B}}_{t} } \right\rangle$ and ${\mathcal{G}}_{2}^{t} = \left\langle {{\mathcal{A}}_{t}^{\prime} ,{\mathcal{B}}_{t}^{\prime} } \right\rangle$ be any two t-${\mathbb{IFG}}$ of ${\mathbb{G}}_{1} = \left\langle {{\mathbb{V}},{\mathbb{E}}} \right\rangle$ and ${\mathbb{G}}_{2} = \left\langle {{{\mathbb{V}}^{\prime}},{{\mathbb{E}}^{\prime}}} \right\rangle ,$ respectively. The Union ${\mathcal{G}}_{1}^{t} \cup {\mathcal{G}}_{2}^{t}$ of two t-${\mathbb{IFG}}$ ${\mathcal{G}}_{1}^{t}$ and ${\mathcal{G}}_{2}^{t}$ is defined as a pair $\left\langle {{\mathcal{A}}_{t} \cup {\mathcal{A}}_{t}^{\prime} ,{\mathcal{B}}_{t} \cup {\mathcal{B}}_{t}^{\prime} } \right\rangle ,$ where ${\mathcal{A}}_{t} \cup {\mathcal{A}}_{t}^{\prime}$ is a t-${\mathbb{IFS}}$ on ${\mathbb{V}} \cup {{\mathbb{V}}^{\prime}}$ and ${\mathcal{B}}_{t} \cup {\mathcal{B}}_{t}^{\prime}$ is a t-${\mathbb{IFS}}$ on ${\mathbb{E}} \cup {{\mathbb{E}}^{\prime}},$ respectively, which satisfy the following conditions:

1. If $$u_{1} \in {\mathbb{V}}$$ and $$u_{1} \notin {{\mathbb{V}}^{\prime}}$$
  - $$\mu_{{{\mathcal{A}}_{t} \cup {\mathcal{A}}_{t}^{\prime} }} \left( {u_{1} } \right) = \mu_{{{\mathcal{A}}_{t} }} \left( {u_{1} } \right)$$
  - $$\sigma_{{{\mathcal{A}}_{t} \cup {\mathcal{A}}_{t}^{\prime} }} \left( {u_{1} } \right) = \sigma_{{{\mathcal{A}}_{t} }} \left( {u_{1} } \right)$$
2. If $$u_{1} \notin {\mathbb{V}}$$ and $$u_{1} \in {{\mathbb{V}}^{\prime}}$$
  - $$\mu_{{{\mathcal{A}}_{t} \cup {\mathcal{A}}_{t}^{\prime} }} \left( {u_{1} } \right) = \mu_{{{\mathcal{A}}_{t}^{\prime} }} \left( {u_{1} } \right)$$
  - $$\sigma_{{{\mathcal{A}}_{t} \cup {\mathcal{A}}_{t}^{\prime} }} \left( {u_{1} } \right) = \sigma_{{{\mathcal{A}}_{t}^{\prime} }} \left( {u_{1} } \right)$$
3. If $$u_{1} \in {\mathbb{V}} \cap {{\mathbb{V}}^{\prime}}$$
  - $$\mu_{{{\mathcal{A}}_{t} \cup {\mathcal{A}}_{t}^{\prime} }} \left( {u_{1} } \right) = max\left\{ {\mu_{{{\mathcal{A}}_{t} }} \left( {u_{1} } \right),\mu_{{{\mathcal{A}}_{t}^{\prime} }} \left( {u_{1} } \right)} \right\}$$
  - $$\sigma_{{{\mathcal{A}}_{t} \cup {\mathcal{A}}_{t}^{\prime} }} \left( {u_{1} } \right) = min\left\{ {\sigma_{{{\mathcal{A}}_{t} }} \left( {u_{1} } \right),\sigma_{{{\mathcal{A}}_{t}^{\prime} }} \left( {u_{1} } \right)} \right\}$$
4. If $$\left( {u_{1} ,w_{1} } \right) \in {\mathbb{E}}$$ and $$\left( {u_{1} ,w_{1} } \right) \notin {{\mathbb{E}}^{\prime}}$$
  - $$\mu_{{{\mathcal{B}}_{t} \cup {\mathcal{B}}_{t}^{\prime} }} \left( {u_{1} ,w_{1} } \right) = \mu_{{{\mathcal{B}}_{t} }} \left( {u_{1} ,w_{1} } \right)$$
  - $$\sigma_{{{\mathcal{B}}_{t} \cup {\mathcal{B}}_{t}^{\prime} }} \left( {u_{1} ,w_{1} } \right) = \sigma_{{{\mathcal{B}}_{t} }} \left( {u_{1} ,w_{1} } \right)$$
5. If $$\left( {u_{1} ,w_{1} } \right) \notin {\mathbb{E}}$$ and $$\left( {u_{1} ,w_{1} } \right) \in {{\mathbb{E}}^{\prime}}$$
  - $$\mu_{{{\mathcal{B}}_{t} \cup {\mathcal{B}}_{t}^{\prime} }} \left( {u_{1} ,w_{1} } \right) = \mu_{{{\mathcal{B}}_{t}^{\prime} }} \left( {u_{1} ,w_{1} } \right)$$
  - $$\sigma_{{{\mathcal{B}}_{t} \cup {\mathcal{B}}_{t}^{\prime} }} \left( {u_{1} ,w_{1} } \right) = \sigma_{{{\mathcal{B}}_{t}^{\prime} }} \left( {u_{1} ,w_{1} } \right)$$
6. If $$\left( {u_{1} ,w_{1} } \right) \in {\mathbb{E}} \cap {{\mathbb{E}}^{\prime}}$$
  - $$\mu_{{{\mathcal{B}}_{t} \cup {\mathcal{B}}_{t}^{\prime} }} \left( {u_{1} ,w_{1} } \right) = max\left\{ {\mu_{{{\mathcal{B}}_{t} }} \left( {u_{1} ,w_{1} } \right),\mu_{{{\mathcal{B}}_{t}^{\prime} }} \left( {u_{1} ,w_{1} } \right)} \right\}$$
  - $$\sigma_{{{\mathcal{B}}_{t} \cup {\mathcal{B}}_{t}^{\prime} }} \left( {u_{1} ,w_{1} } \right) = min\left\{ {\sigma_{{{\mathcal{B}}_{t} }} \left( {u_{1} ,w_{1} } \right),\sigma_{{{\mathcal{B}}_{t}^{\prime} }} \left( {u_{1} ,w_{1} } \right)} \right\}.$$

### Example 9.

**Figure 9:**
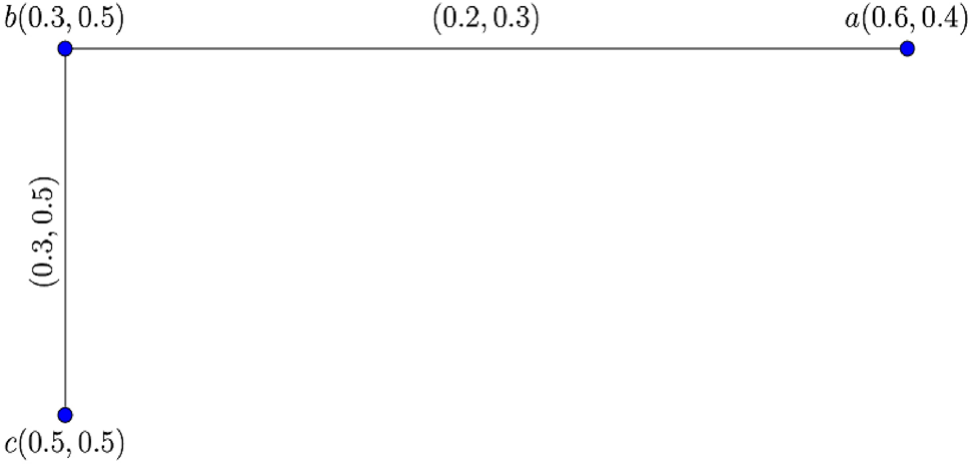
$$0.9 - {\mathbb{IFG}} \,{\mathcal{G}}_{1}^{0.9}$$.

**Figure 10:**
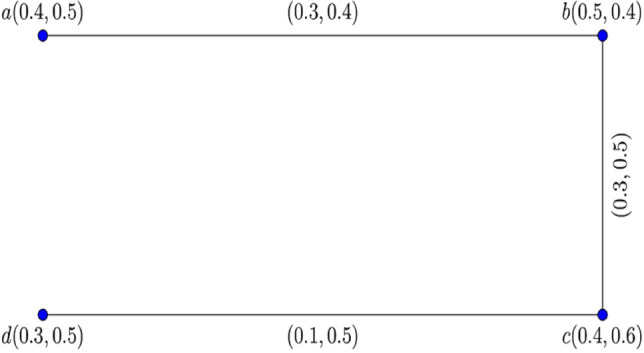
$$0.9 - {\mathbb{IFG}} \,{\mathcal{G}}_{2}^{0.9}$$.

Consider the two $0.9 - {\mathbb{IFG}}$ ${\mathcal{G}}_{1}^{t}$ and ${\mathcal{G}}_{2}^{t}$ as shown in Figs. 9 and 10.

**Figure 11:**
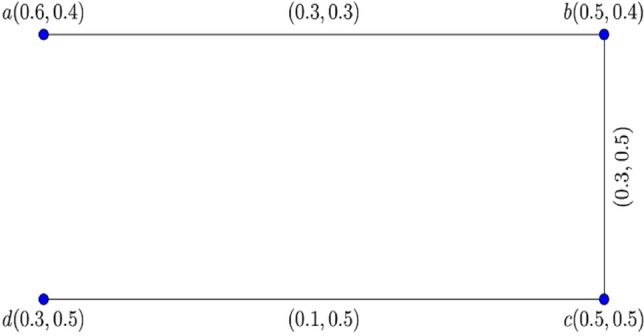
Graphical representation of ${\mathcal{G}}_{1}^{0.9} \cup {\mathcal{G}}_{2}^{0.9}$.

Figure 11 depicts the graphical representation of the union ${\mathcal{G}}_{1}^{0.9} \cup {\mathcal{G}}_{2}^{0.9}$ of two 0.9-${\mathbb{IFG}}$ ${\mathcal{G}}_{1}^{0.9}$ and ${\mathcal{G}}_{2}^{0.9} .$

### Definition 16.

The following formula describes the degree of a vertex $\left( {u_{1} ,w_{1} } \right)$ at a t-${\mathbb{IFG}}$ ${\mathcal{G}}_{1}^{t} \cup {\mathcal{G}}_{2}^{t} :$ For any $\left( {u_{1} ,w_{1} } \right) \in {\mathbb{V}} \times {{\mathbb{V}}^{\prime}}$.

$$deg_{{{\mathcal{G}}_{1}^{t} \cup {\mathcal{G}}_{2}^{t} }} \left( {u_{1} ,w_{1} } \right) = \left( {deg\left( {\mu_{{{\mathcal{B}}_{t} \cup {\mathcal{B}}_{t}^{\prime} }} \left( {\left( {u_{1} ,w_{1} ),(u_{2} ,w_{2} } \right)} \right)} \right),deg\left( {\sigma_{{{\mathcal{B}}_{t} \cup {\mathcal{B}}_{t}^{\prime} }} \left( {\left( {u_{1} ,w_{1} ),(u_{2} ,w_{2} } \right)} \right)} \right)} \right)$$

where

$$\begin{aligned} deg\left( {\mu_{{{\mathcal{B}}_{t} \cup {\mathcal{B}}_{t}^{\prime} }} \left( {\left( {u_{1} ,w_{1} ),(u_{2} ,w_{2} } \right)} \right)} \right) = & \mathop \sum \limits_{{\left( {u_{1} ,w_{1} } \right) \in {\mathbb{E}},{ }(u_{1} ,w_{1} ) \notin {{\mathbb{E}}^{\prime}}}} \mu_{{{\mathcal{B}}_{t} }} \left( {u_{1} ,w_{1} } \right) \\ & + \mathop \sum \limits_{{(u_{1} ,w_{1} ) \notin {\mathbb{E}},{ }\left( {u_{1} ,w_{1} } \right) \in {{\mathbb{E}}^{\prime}}}} \mu_{{{\mathcal{B}}_{t}^{\prime} }} \left( {u_{1} ,w_{1} } \right) \\ & + \mathop \sum \limits_{{\left( {u_{1} ,w_{1} } \right) \in {\mathbb{E}} \cap {{\mathbb{E}}^{\prime}}}} max\left\{ {\mu_{{{\mathcal{B}}_{t} }} \left( {u_{1} ,w_{1} } \right),\mu_{{{\mathcal{B}}_{t}^{\prime} }} \left( {u_{1} ,w_{1} } \right)} \right\} \\ \end{aligned}$$

and

$$\begin{aligned} deg\left( {\sigma_{{{\mathcal{B}}_{t} \cup {\mathcal{B}}_{t}^{\prime} }} \left( {\left( {u_{1} ,w_{1} ),(u_{2} ,w_{2} } \right)} \right)} \right) = & \mathop \sum \limits_{{\left( {u_{1} ,w_{1} } \right) \in {\mathbb{E}},{ }(u_{1} ,w_{1} ) \notin {{\mathbb{E}}^{\prime}}}} \sigma_{{{\mathcal{B}}_{t} }} \left( {u_{1} ,w_{1} } \right) \\ & + \mathop \sum \limits_{{(u_{1} ,w_{1} ) \notin {\mathbb{E}},{ }\left( {u_{1} ,w_{1} } \right) \in {{\mathbb{E}}^{\prime}}}} \sigma_{{{\mathcal{B}}_{t}^{\prime} }} \left( {u_{1} ,w_{1} } \right) \\ & + \mathop \sum \limits_{{\left( {u_{1} ,w_{1} } \right) \in {\mathbb{E}} \cap {{\mathbb{E}}^{\prime}}}} min\left\{ {\sigma_{{{\mathcal{B}}_{t} }} \left( {\left( {u_{1} ,w_{1} } \right),\mu_{{{\mathcal{B}}_{t}^{\prime} }} \left( {u_{1} ,w_{1} } \right)} \right)} \right\} \\ \end{aligned}$$

### Proposition 5.

The union of two t-$${\mathbb{IFG}}$$ is also a t-$${\mathbb{IFG}}$$$$.$$

### Definition 17.

Let ${\mathcal{G}}_{1}^{t} = \left\langle {{\mathcal{A}}_{t} ,{\mathcal{B}}_{t} } \right\rangle$ and ${\mathcal{G}}_{2}^{t} = \left\langle {{\mathcal{A}}_{t}^{\prime} ,{\mathcal{B}}_{t}^{\prime} } \right\rangle$ be any two t-${\mathbb{IFG}}$. The join ${\mathcal{G}}_{1}^{t} + {\mathcal{G}}_{2}^{t}$ of two t-${\mathbb{IFG}}$ ${\mathcal{G}}_{1}^{t} = \left\langle {{\mathcal{A}}_{t} ,{\mathcal{B}}_{t} } \right\rangle$ and ${\mathcal{G}}_{2}^{t} = \left\langle {{\mathcal{A}}_{t}^{\prime} ,{\mathcal{B}}_{t}^{\prime} } \right\rangle$ is defined as $\left\langle {{\mathcal{A}}_{t} + {\mathcal{A}}_{t}^{\prime} ,{\mathcal{B}}_{t} + {\mathcal{B}}_{t}^{\prime} } \right\rangle ,$ where ${\mathcal{A}}_{t} + {\mathcal{A}}_{t}^{\prime}$ is a t-${\mathbb{IFS}}$ on ${\mathbb{V}} \cup {{\mathbb{V}}^{\prime}}$ and ${\mathcal{B}}_{t} + {\mathcal{B}}_{t}^{\prime}$ is a t-${\mathbb{IFS}}$ on ${\mathbb{E}} \cup {{\mathbb{E}}^{\prime}} \cup {{\mathbb{E}}^{\prime\prime}}({{\mathbb{E}}^{\prime\prime}}$ is the set of all edges joining the vertices of ${\mathbb{V}}$ and ${{\mathbb{V}}^{\prime}})$ respectively, which satisfies the following conditions:

1. If $$u_{1} \in {\mathbb{V}}$$ and $$u_{1} \notin {{\mathbb{V}}^{\prime}}$$
  - $$\mu_{{{\mathcal{A}}_{t} + {\mathcal{A}}_{t}^{\prime} }} \left( {u_{1} } \right) = \mu_{{{\mathcal{A}}_{t} }} \left( {u_{1} } \right)$$
  - $$\sigma_{{{\mathcal{A}}_{t} + {\mathcal{A}}_{t}^{\prime} }} \left( {u_{1} } \right) = \sigma_{{{\mathcal{A}}_{t} }} \left( {u_{1} } \right)$$
2. If $$u_{1} \notin {\mathbb{V}}$$ and $$u_{1} \in {{\mathbb{V}}^{\prime}}$$
  - $$\mu_{{{\mathcal{A}}_{t} + {\mathcal{A}}_{t}^{\prime} }} \left( {u_{1} } \right) = \mu_{{{\mathcal{A}}_{t}^{\prime} }} \left( {u_{1} } \right)$$
  - $$\sigma_{{{\mathcal{A}}_{t} + {\mathcal{A}}_{t}^{\prime} }} \left( {u_{1} } \right) = \sigma_{{{\mathcal{A}}_{t}^{\prime} }} \left( {u_{1} } \right)$$
3. If $$u_{1} \in {\mathbb{V}} \cap {{\mathbb{V}}^{\prime}}$$
  - $$\mu_{{{\mathcal{A}}_{t} + {\mathcal{A}}_{t}^{\prime} }} \left( {u_{1} } \right) = max\left\{ {\mu_{{{\mathcal{A}}_{t} }} \left( {u_{1} } \right),\mu_{{{\mathcal{A}}_{t}^{\prime} }} \left( {u_{1} } \right)} \right\}$$
  - $$\sigma_{{{\mathcal{A}}_{t} + {\mathcal{A}}_{t}^{\prime} }} \left( {u_{1} } \right) = min\left\{ {\sigma_{{{\mathcal{A}}_{t} }} \left( {u_{1} } \right),\sigma_{{{\mathcal{A}}_{t}^{\prime} }} \left( {u_{1} } \right)} \right\}$$
4. If $$\left( {u_{1} ,w_{1} } \right) \in {\mathbb{E}}$$ and $$\left( {u_{1} ,w_{1} } \right) \notin {{\mathbb{E}}^{\prime}}$$
  - $$\mu_{{{\mathcal{B}}_{t} + {\mathcal{B}}_{t}^{\prime} }} \left( {u_{1} ,w_{1} } \right) = \mu_{{{\mathcal{B}}_{t} }} \left( {u_{1} ,w_{1} } \right)$$
  - $$\sigma_{{{\mathcal{B}}_{t} + {\mathcal{B}}_{t}^{\prime} }} \left( {u_{1} ,w_{1} } \right) = \sigma_{{{\mathcal{B}}_{t} }} \left( {u_{1} ,w_{1} } \right)$$
5. If $$\left( {u_{1} ,w_{1} } \right) \notin {\mathbb{E}}$$ and $$\left( {u_{1} ,w_{1} } \right) \in {{\mathbb{E}}^{\prime}}$$
  - $$\mu_{{{\mathcal{B}}_{t} + {\mathcal{B}}_{t}^{\prime} }} \left( {u_{1} ,w_{1} } \right) = \mu_{{{\mathcal{B}}_{t}^{\prime} }} \left( {u_{1} ,w_{1} } \right)$$
  - $$\sigma_{{{\mathcal{B}}_{t} + {\mathcal{B}}_{t}^{\prime} }} \left( {u_{1} ,w_{1} } \right) = \sigma_{{{\mathcal{B}}_{t}^{\prime} }} \left( {u_{1} ,w_{1} } \right)$$
6. If $$\left( v \right) \in {\mathbb{E}} \cap {{\mathbb{E}}^{\prime}}$$
  - $$\mu_{{{\mathcal{B}}_{t} + {\mathcal{B}}_{t}^{\prime} }} \left( {u_{1} ,w_{1} } \right) = max\left\{ {\mu_{{{\mathcal{B}}_{t} }} \left( {u_{1} ,w_{1} } \right),\mu_{{{\mathcal{B}}_{t}^{\prime} }} \left( {u_{1} ,w_{1} } \right)} \right\}$$
  - $$\sigma_{{{\mathcal{B}}_{t} + {\mathcal{B}}_{t}^{\prime} }} \left( {u_{1} ,w_{1} } \right) = min\left\{ {\sigma_{{{\mathcal{B}}_{t} }} \left( {u_{1} ,w_{1} } \right),\sigma_{{{\mathcal{B}}_{t}^{\prime} }} \left( {u_{1} ,w_{1} } \right)} \right\}.$$
7. If $$\left( {u_{1} ,w_{1} } \right) \in {{\mathbb{E}}^{\prime\prime}}$$
  - $$\mu_{{{\mathcal{B}}_{t} + {\mathcal{B}}_{t}^{\prime} }} \left( {u_{1} ,w_{1} } \right) = min\left\{ {\mu_{{{\mathcal{A}}_{t} }} \left( {u_{1} } \right),\mu_{{{\mathcal{A}}_{t}^{\prime} }} \left( {w_{1} } \right)} \right\}$$
  - $$\sigma_{{{\mathcal{B}}_{t} + {\mathcal{B}}_{t}^{\prime} }} \left( {u_{1} ,w_{1} } \right) = max\left\{ {\sigma_{{{\mathcal{A}}_{t} }} \left( {u_{1} } \right),\sigma_{{{\mathcal{A}}_{t}^{\prime} }} \left( {w_{1} } \right)} \right\}$$

### Example 10.

**Figure 12:**
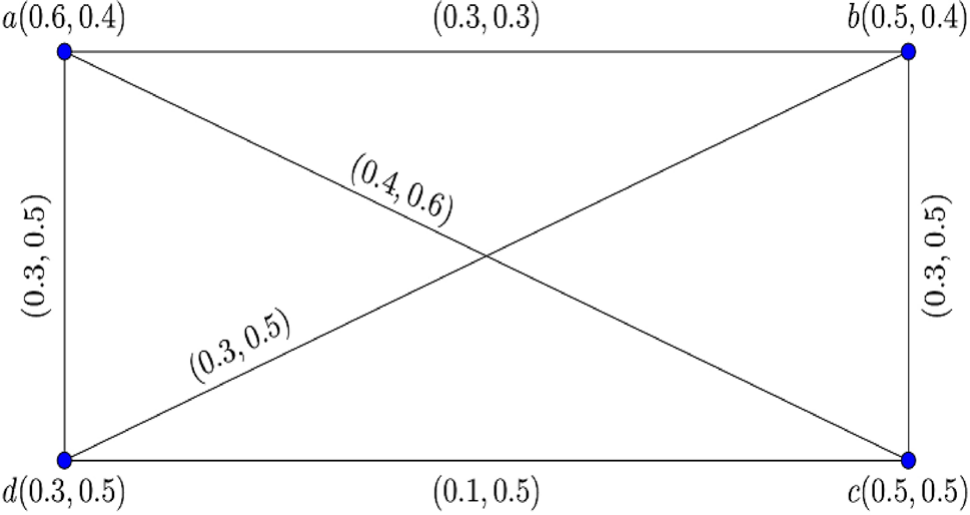
Graphical representation of ${\mathcal{G}}_{1}^{0.9} + {\mathcal{G}}_{2}^{0.9}$.

From Example 9, the graphical representation of $0.9 - {\mathbb{IFG}}$ ${\mathcal{G}}_{1}^{t} + {\mathcal{G}}_{2}^{t}$ of ${\mathcal{G}}_{1}^{t}$ and ${\mathcal{G}}_{2}^{t}$ as shown in Fig. 12.

### Definition 18.

Let ${\mathcal{G}}_{1}^{t}$ and ${\mathcal{G}}_{2}^{t}$ be any two $t - {\mathbb{IFG}}$*.* The degree of a vertex in t-${\mathbb{IFG}}$ ${\mathcal{G}}_{1}^{t} + {\mathcal{G}}_{2}^{t}$ is defined as follows: for any $\left( {u_{1} ,w_{1} } \right) \in {\mathbb{V}} \times {{\mathbb{V}}^{\prime}}$.

$$deg_{{{\mathcal{G}}_{1}^{t} + {\mathcal{G}}_{2}^{t} }} \left( {u_{1} ,w_{1} } \right) = \left( {deg\left( {\mu_{{{\mathcal{B}}_{t} + {\mathcal{B}}_{t}^{\prime} }} \left( {\left( {u_{1} ,w_{1} ),(u_{2} ,w_{2} } \right)} \right)} \right),deg\left( {\sigma_{{{\mathcal{B}}_{t} + {\mathcal{B}}_{t}^{\prime} }} \left( {\left( {u_{1} ,w_{1} ),(u_{2} ,w_{2} } \right)} \right)} \right)} \right)$$

where

$$\begin{aligned} deg\left( {\mu_{{{\mathcal{B}}_{t} + {\mathcal{B}}_{t}^{\prime} }} \left( {\left( {u_{1} ,w_{1} ),(u_{2} ,w_{2} } \right)} \right)} \right) = & \mathop \sum \limits_{{u_{1} \in {\mathbb{V}} \cup {{\mathbb{V}}^{\prime}}}} \mu_{{{\mathcal{A}}_{t} \cup {\mathcal{A}}_{t}^{\prime} }} \left( {u_{1} } \right) + \mathop \sum \limits_{{\left( {u_{1} ,w_{1} } \right) \in {\mathbb{E}} \cap {{\mathbb{E}}^{\prime}}}} \mu_{{{\mathcal{B}}_{t} \cup {\mathcal{B}}_{t}^{\prime} }} \left( {u_{1} ,w_{1} } \right) \\ & + \mathop \sum \limits_{{(u_{1} ,w_{1} ) \in {{\mathbb{E}}^{\prime\prime}}}} min\left\{ {\mu_{{{\mathcal{A}}_{t} }} \left( {u_{1} } \right),\mu_{{{\mathcal{A}}_{t}^{\prime} }} \left( {w_{1} } \right)} \right\} \\ \end{aligned}$$

and

$$\begin{aligned} deg\left( {\sigma_{{{\mathcal{B}}_{t} + {\mathcal{B}}_{t}^{\prime} }} \left( {\left( {u_{1} ,w_{1} ),(u_{2} ,w_{2} } \right)} \right)} \right) = & \mathop \sum \limits_{{u_{1} \in {\mathbb{V}} \cup {{\mathbb{V}}^{\prime}}}} \sigma_{{{\mathcal{A}}_{t} \cup {\mathcal{A}}_{t}^{\prime} }} \left( {u_{1} } \right) + \mathop \sum \limits_{{\left( {u_{1} ,w_{1} } \right) \in {\mathbb{E}} \cap {{\mathbb{E}}^{\prime}}}} \sigma_{{{\mathcal{B}}_{t} \cup {\mathcal{B}}_{t}^{\prime} }} \left( {u_{1} ,w_{1} } \right) \\ & + \mathop \sum \limits_{{\left( {u_{1} ,w_{1} } \right) \in {{\mathbb{E}}^{\prime\prime}}}} max\left\{ {\sigma_{{{\mathcal{A}}_{t} }} \left( {u_{1} } \right),\sigma_{{{\mathcal{A}}_{t}^{\prime} }} \left( {w_{1} } \right)} \right\} \\ \end{aligned}$$

### Proposition 6.

The join of two t-$${\mathbb{IFG}}$$ is also a t-$${\mathbb{IFG}}$$$$.$$

### Theorem 2.

Let ${\mathcal{G}}_{1}^{t} = \left\langle {{\mathcal{A}}_{t} ,{\mathcal{B}}_{t} } \right\rangle$ and ${\mathcal{G}}_{2}^{t} = \left\langle {{\mathcal{A}}_{t}^{\prime} ,{\mathcal{B}}_{t}^{\prime} } \right\rangle$ be t-${\mathbb{IFG}}$ of ${{\mathbb{G}}^{\prime}}$ and ${{\mathbb{G}}^{\prime\prime}},$ respectively and let ${\mathbb{V}} \cap {{\mathbb{V}}^{\prime}} = \emptyset .$ The union ${\mathcal{G}}_{1}^{t} \cup {\mathcal{G}}_{2}^{t} = \left\langle {{\mathcal{A}}_{t} \cup {\mathcal{A}}_{t}^{\prime} ,{\mathcal{B}}_{t} \cup {\mathcal{B}}_{t}^{\prime} } \right\rangle$ is a t-${\mathbb{IFG}}$ of ${\mathbb{G}} = {{\mathbb{G}}^{\prime}} \cup {{\mathbb{G}}^{\prime\prime}}$ if and only if ${\mathcal{G}}_{1}^{t}$ and ${\mathcal{G}}_{2}^{t}$ are t-${\mathbb{IFG}}$ of ${{\mathbb{G}}^{\prime}}$ and ${{\mathbb{G}}^{\prime\prime}},$ respectively.

### Proof.

Suppose that ${\mathcal{G}}_{1}^{t} \cup {\mathcal{G}}_{2}^{t}$ is a t-${\mathbb{IFG}}$*.* Let $\left( {u_{1} ,w_{1} } \right) \in {\mathbb{E}},$ $\left( {u_{1} ,w_{1} } \right) \notin {{\mathbb{E}}^{\prime}}$ and $u_{1} ,w_{1} \in {\mathbb{V}} - {{\mathbb{V}}^{\prime}}$*.*

Consider

$$\begin{aligned} \mu_{{{\mathcal{B}}_{t} }} \left( {u_{1} ,w_{1} } \right) = & \,\mu_{{B_{t} \cap {\mathcal{B}}_{t}^{\prime} }} \left( {u_{1} ,w_{1} } \right) \\ \le &\, min\left\{ {\mu_{{{\mathcal{A}}_{t} \cap {\mathcal{A}}_{t}^{\prime} }} \left( {u_{1} } \right),\mu_{{{\mathcal{A}}_{t} \cap {\mathcal{A}}_{t}^{\prime} }} \left( {w_{1} } \right)} \right\} \\ = &\, min\left\{ {\mu_{{{\mathcal{A}}_{t} }} \left( {u_{1} } \right),\mu_{{{\mathcal{A}}_{t} }} \left( {w_{1} } \right)} \right\} \\ \end{aligned}$$

Consequently $$\mu_{{{\mathcal{B}}_{t} }} \left( {u_{1} ,w_{1} } \right) \le min\left\{ {\mu_{{{\mathcal{A}}_{t} }} \left( {u_{1} } \right),\mu_{{{\mathcal{A}}_{t} }} \left( {w_{1} } \right)} \right\}.$$

Also

$$\begin{aligned} \sigma_{{{\mathcal{B}}_{t} }} \left( {u_{1} ,w_{1} } \right) = &\, \sigma_{{B_{t} \cap {\mathcal{B}}_{t}^{\prime} }} \left( {u_{1} ,w_{1} } \right) \\ \le &\, max\left\{ {\sigma_{{{\mathcal{A}}_{t} \cap {\mathcal{A}}_{t}^{\prime} }} \left( {u_{1} } \right),\sigma_{{{\mathcal{A}}_{t} \cap {\mathcal{A}}_{t}^{\prime} }} \left( {w_{1} } \right)} \right\} \\ = &\, max\left\{ {\sigma_{{{\mathcal{A}}_{t} }} \left( {u_{1} } \right),\sigma_{{{\mathcal{A}}_{t} }} \left( {w_{1} } \right)} \right\} \\ \end{aligned}$$

Consequently $$\sigma_{{{\mathcal{B}}_{t} }} \left( {u_{1} ,w_{1} } \right) \le max\left\{ {\sigma_{{{\mathcal{A}}_{t} }} \left( {u_{1} } \right),\sigma_{{{\mathcal{A}}_{t} }} \left( {w_{1} } \right)} \right\}.$$

This shows that $${\mathcal{G}}_{1}^{t} = \left\langle {{\mathcal{A}}_{t} ,{\mathcal{B}}_{t} } \right\rangle$$ is a t-$${\mathbb{IFG}}$$*.* In the same way, we obtain that $${\mathcal{G}}_{2}^{t} = \left\langle {{\mathcal{A}}_{t}^{\prime} ,{\mathcal{B}}_{t}^{\prime} } \right\rangle$$ is a t-$${\mathbb{IFG}}$$ of $${{\mathbb{G}}^{\prime\prime}}.$$ Conversely, suppose that $${\mathcal{G}}_{1}^{t}$$ and $${\mathcal{G}}_{2}^{t}$$ are t-$${\mathbb{IFG}}$$$$.$$ We know that the union of two t-$${\mathbb{IFG}}$$ is a t-$${\mathbb{IFG}}$$. Thus, $${\mathcal{G}}_{1}^{t} \cup {\mathcal{G}}_{2}^{t}$$ is a t-$${\mathbb{IFG}}$$$$.$$

### Theorem 3.

Let ${\mathcal{G}}_{1}^{t} = \left\langle {{\mathcal{A}}_{t} ,{\mathcal{B}}_{t} } \right\rangle$ and ${\mathcal{G}}_{2}^{t} = \left\langle {{\mathcal{A}}_{t}^{\prime} ,{\mathcal{B}}_{t}^{\prime} } \right\rangle$ be t-${\mathbb{IFG}}$ of ${{\mathbb{G}}^{\prime}}$ and ${{\mathbb{G}}^{\prime\prime}}$ respectively and let ${\mathbb{V}} \cap {{\mathbb{V}}^{\prime}} = \emptyset .$ Then join ${\mathcal{G}}_{1}^{t} + {\mathcal{G}}_{2}^{t} = \left\langle {{\mathcal{A}}_{t} + {\mathcal{A}}_{t}^{\prime} ,{\mathcal{B}}_{t} + {\mathcal{B}}_{t}^{\prime} } \right\rangle$ is a t-${\mathbb{IFG}}$ of ${\mathbb{G}} = {{\mathbb{G}}^{\prime}} \cup {{\mathbb{G}}^{\prime\prime}}$ if and only if ${\mathcal{G}}_{1}^{t}$ and ${\mathcal{G}}_{2}^{t}$ are t-${\mathbb{IFG}}$ of ${{\mathbb{G}}^{\prime}}$ and ${{\mathbb{G}}^{\prime\prime}}$ respectively.

### Proof.

The proof for this is similar to the proof presented in Theorem 2.

## Isomorphism of t-intuitionistic fuzzy graphs

This section introduces the concepts of homomorphism and isomorphism of t-${\mathbb{IFG}}$ and explores the essential properties of these ideas.

### Definition 19.

Let ${\mathcal{G}}_{1}^{t} = \left\langle {{\mathcal{A}}_{t} ,{\mathcal{B}}_{t} } \right\rangle$ and ${\mathcal{G}}_{2}^{t} = \left\langle {{\mathcal{A}}_{t}^{\prime} ,{\mathcal{B}}_{t}^{\prime} } \right\rangle$ be t-${\mathbb{IFG}}$ of ${{\mathbb{G}}^{\prime}}\left\langle {{\mathbb{V}},{\mathbb{E}}} \right\rangle =$ and ${{\mathbb{G}}^{\prime\prime}} = \left\langle {{{\mathbb{V}}^{\prime}} ,{{\mathbb{E}}^{\prime}}} \right\rangle$ respectively. A homomorphism $\theta$ from t-${\mathbb{IFG}}$ ${\mathcal{G}}_{1}^{t}$ to ${\mathcal{G}}_{2}^{t}$ is a mapping $\theta :{\mathbb{V}} \to {{\mathbb{V}}^{\prime}},$ satisfying the following conditions:

1. $$\mu_{{{\mathcal{A}}_{t} }} \left( {u_{1} } \right) \le \mu_{{{\mathcal{A}}_{t}^{\prime} }} \left( {\theta \left( {u_{1} } \right)} \right)$$ and $$\sigma_{{{\mathcal{A}}_{t} }} \left( {u_{1} } \right) \le \sigma_{{{\mathcal{A}}_{t}^{\prime} }} \left( {\theta \left( {u_{1} } \right)} \right)$$, $$\forall u_{1} \in {\mathbb{V}}$$
2. $$\mu_{{{\mathcal{B}}_{t} }} \left( {u_{1} ,w_{1} } \right) \le \mu_{{{\mathcal{B}}_{t}^{\prime} }} \left( {\theta \left( {u_{1} } \right),\theta \left( {w_{1} } \right)} \right)$$ and $$\sigma_{{{\mathcal{B}}_{t} }} \left( {u_{1} ,w_{1} } \right) \le \sigma_{{{\mathcal{B}}_{t}^{\prime} }} \left( {\theta \left( {u_{1} } \right),\theta \left( {w_{1} } \right)} \right)$$, $$\forall (u_{1} ,w_{1} ) \in {\mathbb{E}}.$$

### Definition 20.

A weak isomorphism $\theta$ from t-${\mathbb{IFG}}$ ${\mathcal{G}}_{1}^{t}$ to ${\mathcal{G}}_{2}^{t}$ is a bijective mapping $\theta :{\mathbb{V}} \to {{\mathbb{V}}^{\prime}},$ which meets the following conditions:

$$\mu_{{{\mathcal{A}}_{t} }} \left( {u_{1} } \right) = \mu_{{{\mathcal{A}}_{t}^{\prime} }} \left( {\theta \left( {u_{1} } \right)} \right)\quad {\text{and}}\quad \sigma_{{{\mathcal{A}}_{t} }} \left( {u_{1} } \right) = \sigma_{{{\mathcal{A}}_{t}^{\prime} }} \left( {\theta \left( {u_{1} } \right)} \right),\;\forall u_{1} \in {\mathbb{V}}.$$

### Definition 21.

Let ${\mathcal{G}}_{1}^{t} = \left\langle {{\mathcal{A}}_{t} ,{\mathcal{B}}_{t} } \right\rangle$ and ${\mathcal{G}}_{2}^{t} = \left\langle {{\mathcal{A}}_{t}^{\prime} ,{\mathcal{B}}_{t}^{\prime} } \right\rangle$ be t-${\mathbb{IFG}}$ of ${{\mathbb{G}}^{\prime}}\left\langle {{\mathbb{V}},{\mathbb{E}}} \right\rangle =$ and ${{\mathbb{G}}^{\prime\prime}} = \left\langle {{{\mathbb{V}}^{\prime}} ,{{\mathbb{E}}^{\prime}}} \right\rangle$ respectively. A bijective mapping $\theta :{\mathbb{V}} \to {{\mathbb{V}}^{\prime}}$ is a strong co-isomorphism if it satisfies the below conditions:

1. $$\mu_{{{\mathcal{A}}_{t} }} \left( {u_{1} } \right) \le \mu_{{{\mathcal{A}}_{t}^{\prime} }} \left( {\theta \left( {u_{1} } \right)} \right)$$ and $$\sigma_{{{\mathcal{A}}_{t} }} \left( {u_{1} } \right) \le \sigma_{{{\mathcal{A}}_{t}^{\prime} }} \left( {\theta \left( {u_{1} } \right)} \right)$$*,*$$\forall u_{1} \in {\mathbb{V}}$$
2. $$\mu_{{{\mathcal{B}}_{t} }} \left( {u_{1} ,w_{1} } \right) \le \mu_{{{\mathcal{B}}_{t}^{\prime} }} \left( {\theta \left( {u_{1} } \right),\theta \left( {w_{1} } \right)} \right)$$ and $$\sigma_{{{\mathcal{B}}_{t} }} \left( {u_{1} ,w_{1} } \right) \le \sigma_{{{\mathcal{B}}_{t}^{\prime} }} \left( {\theta \left( {u_{1} } \right),\theta \left( {w_{1} } \right)} \right)$$, $$\forall \left( {u_{1} ,w_{1} } \right) \in {\mathbb{E}}$$
3. $$\mu_{{{\mathcal{B}}_{t} }} \left( {u_{1} ,w_{1} } \right) = \mu_{{{\mathcal{B}}_{t}^{\prime} }} \left( {\theta \left( {u_{1} } \right),\theta \left( {w_{1} } \right)} \right)$$ and $$\sigma_{{{\mathcal{B}}_{t} }} \left( {u_{1} ,w_{1} } \right) = \sigma_{{{\mathcal{B}}_{t}^{\prime} }} \left( {\theta \left( {u_{1} } \right),\theta \left( {w_{1} } \right)} \right)$$, $$\forall \left( {u_{1} ,w_{1} } \right) \in {\mathbb{E}}.$$

### Definition 22.

An isomorphism between t-${\mathbb{IFG}}$s ${\mathcal{G}}_{1}^{t} = \left\langle {{\mathcal{A}}_{t} ,{\mathcal{B}}_{t} } \right\rangle$ and ${\mathcal{G}}_{2}^{t} = \left\langle {{\mathcal{A}}_{t}^{\prime} ,{\mathcal{B}}_{t}^{\prime} } \right\rangle$ is a bijective homomorphism mapping $\theta :{\mathbb{V}} \to {{\mathbb{V}}^{\prime}}$ (written as ${\mathcal{G}}_{1}^{t} \approx {\mathcal{G}}_{2}^{t} )$ which satisfies the following conditions:

1. $$\mu_{{{\mathcal{A}}_{t} }} \left( {u_{1} } \right) = \mu_{{{\mathcal{A}}_{t}^{\prime} }} \left( {\theta \left( {u_{1} } \right)} \right)$$ and $$\sigma_{{{\mathcal{A}}_{t} }} \left( {u_{1} } \right) = \sigma_{{{\mathcal{A}}_{t}^{\prime} }} \left( {\theta \left( {u_{1} } \right)} \right)$$, $$\forall u_{1} \in {\mathbb{V}}$$
2. $$\mu_{{{\mathcal{B}}_{t} }} \left( {u_{1} ,w_{1} } \right) = \mu_{{{\mathcal{B}}_{t}^{\prime} }} \left( {\theta \left( {u_{1} } \right),\theta \left( {w_{1} } \right)} \right)$$ and $$\sigma_{{{\mathcal{B}}_{t} }} \left( {u_{1} ,w_{1} } \right) = \sigma_{{{\mathcal{B}}_{t}^{\prime} }} \left( {\theta \left( {u_{1} } \right),\theta \left( {w_{1} } \right)} \right)$$, $$\forall \left( {u_{1} ,w_{1} } \right) \in {\mathbb{E}}.$$

### Example 11.

**Figure 13:**
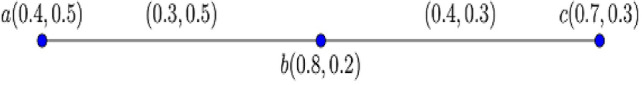
$$0.8 - {\mathbb{IFG}}$$ $${\mathcal{G}}_{1}^{0.8}$$.

**Figure 14:**
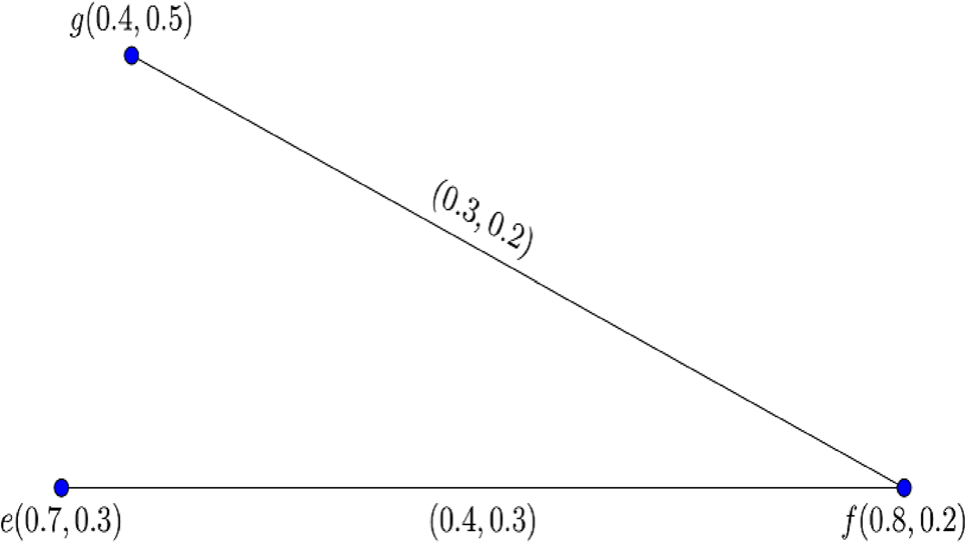
$$0.8 - {\mathbb{IFG}} \,{\mathcal{G}}_{2}^{0.8}$$.

Consider the two $0.8 - {\mathcal{G}}_{1}^{t}$ and ${\mathcal{G}}_{2}^{t}$ as shown in Figs. 13 and 14.

The mapping $\zeta :{\mathbb{V}} \to {{\mathbb{V}}^{\prime}}$ is defined by $\zeta \left( a \right) = g,\zeta \left( b \right) = f$ and $\zeta \left( c \right) = e.$ Given Definition (22), we have ${\mathcal{G}}_{1}^{0.8} \approx {\mathcal{G}}_{2}^{0.8} .$

### Theorem 4.

An isomorphism between t-${\mathbb{IFG}}$ is an equivalence relation.

### Proof.

Reflexivity and symmetry are obvious. Let $\varphi :{\mathbb{V}} \to {{\mathbb{V}}^{\prime}}$ and $\theta :{{\mathbb{V}}^{\prime}} \to {{\mathbb{V}}^{\prime\prime}}$ be the isomorphisms of ${\mathcal{G}}_{1}^{t}$ onto ${\mathcal{G}}_{2}^{t}$ and ${\mathcal{G}}_{2}^{t}$ onto ${\mathcal{G}}_{3}^{t}$ respectively.

Then $\theta \circ \varphi :{\mathbb{V}} \to {{\mathbb{V}}^{\prime\prime}}$ is a bijective map from ${\mathbb{V}}$ to ${{\mathbb{V}}^{\prime\prime}}$ is defined as:

$$\left( {\theta \circ \varphi } \right)\left( {u_{1} } \right) = \theta \left( {\varphi \left( {u_{1} } \right)} \right),\forall u_{1} \in {\mathbb{V}}.$$

Since a map $\varphi :{\mathbb{V}} \to {{\mathbb{V}}^{\prime}}$ defined by $\varphi \left( {u_{1} } \right) = w_{1} ,\forall u_{1} \in {\mathbb{V}}$ is an isomorphism.

In view of Definition (22), we have

1 $$\mu_{{{\mathcal{A}}_{t} }} \left( {u_{1} } \right) = \mu_{{{\mathcal{A}}_{t}^{\prime} }} \left( {\varphi \left( {u_{1} } \right)} \right) = \mu_{{{\mathcal{A}}_{t}^{\prime} }} \left( {w_{1} } \right),\forall u_{1} \in {\mathbb{V}}$$

2 $$\sigma_{{{\mathcal{A}}_{t} }} \left( {u_{1} } \right) = \sigma_{{{\mathcal{A}}_{t}^{\prime} }} \left( {\varphi \left( {u_{1} } \right)} \right) = \sigma_{{{\mathcal{A}}_{t}^{\prime} }} \left( {w_{1} } \right),\forall u_{1} \in {\mathbb{V}}$$

and

3 $$\mu_{{{\mathcal{B}}_{t} }} \left( {u_{1} ,u_{2} } \right) = \mu_{{{\mathcal{B}}_{t}^{\prime} }} \left( {\varphi \left( {u_{1} } \right),\varphi \left( {u_{2} } \right)} \right) = \mu_{{{\mathcal{B}}_{t}^{\prime} }} \left( {w_{1} ,w_{2} } \right),\forall \left( {u_{1} ,u_{2} } \right) \in {\mathbb{E}}$$

4 $$\sigma_{{{\mathcal{B}}_{t} }} \left( {u_{1} ,u_{2} } \right) = \sigma_{{{\mathcal{B}}_{t}^{\prime} }} \left( {\varphi \left( {u_{1} } \right),\varphi \left( {u_{2} } \right)} \right) = \sigma_{{{\mathcal{B}}_{t}^{\prime} }} \left( {w_{1} ,w_{2} } \right),\forall \left( {u_{1} ,u_{2} } \right) \in {\mathbb{E}}.$$

In the same way, we obtained that

5 $$\mu_{{{\mathcal{A}}_{t}^{\prime} }} \left( {w_{1} } \right) = \mu_{{{\mathcal{A}}_{t}^{^{\prime\prime}} }} \left( {v_{1} } \right),\forall w_{1} \in {{\mathbb{V}}^{\prime}}$$

6 $$\sigma_{{{\mathcal{A}}_{t}^{\prime} }} \left( {w_{1} } \right) = \sigma_{{{\mathcal{A}}_{t}^{^{\prime\prime}} }} \left( {v_{1} } \right),\forall w_{1} \in {{\mathbb{V}}^{\prime}}$$

*and*

7 $$\mu_{{{\mathcal{B}}_{t}^{\prime} }} \left( {w_{1} ,w_{2} } \right) = \mu_{{{\mathcal{B}}_{t}^{^{\prime\prime}} }} \left( {v_{1} ,v_{2} } \right),\forall (w_{1} ,w_{2} ) \in {{\mathbb{E}}^{\prime}}$$

8 $$\sigma_{{{\mathcal{B}}_{t}^{\prime} }} \left( {w_{1} ,w_{2} } \right) = \sigma_{{{\mathcal{B}}_{t}^{^{\prime\prime}} }} \left( {v_{1} ,v_{2} } \right),\forall (w_{1} ,w_{2} ) \in {{\mathbb{E}}^{\prime}}.$$

By using the relations (1), (5) and $\varphi \left( {u_{1} } \right) = w_{1} ,u_{1} \in {\mathbb{V}},$ we have

$$\mu_{{{\mathcal{A}}_{t} }} \left( {u_{1} } \right) = \mu_{{{\mathcal{A}}_{t}^{\prime} }} \left( {\varphi \left( {u_{1} } \right)} \right) = \mu_{{{\mathcal{A}}_{t}^{\prime} }} \left( {w_{1} } \right) = \mu_{{{\mathcal{A}}_{t}^{^{\prime\prime}} }} \left( {\theta (w_{1} )} \right) = \mu_{{{\mathcal{A}}_{t}^{^{\prime\prime}} }} \left( {\theta \left( {\varphi \left( {u_{1} } \right)} \right)} \right).$$

Similarly, we obtain that $\sigma_{{{\mathcal{A}}_{t} }} \left( {u_{1} } \right) = \sigma_{{{\mathcal{A}}_{t}^{^{\prime\prime}} }} \left( {\theta \left( {\varphi \left( {u_{1} } \right)} \right)} \right).$

When relations (3) and (7) are applied, the outcome is that:

$$\mu_{{{\mathcal{B}}_{t} }} \left( {u_{1} ,u_{2} } \right) = \mu_{{{\mathcal{B}}_{t}^{\prime} }} \left( {w_{1} ,w_{2} } \right) = \mu_{{{\mathcal{B}}_{t}^{^{\prime\prime}} }} \left( {\theta (w_{1} ),\theta (w_{2} )} \right) = \mu_{{{\mathcal{B}}_{t}^{^{\prime\prime}} }} \left( {\theta (\varphi (u_{1} )),\theta (\varphi (u_{2} ))} \right).$$

Similarly, we find that $\mu_{{{\mathcal{B}}_{t} }} \left( {u_{1} ,u_{2} } \right) = \mu_{{{\mathcal{B}}_{t}^{^{\prime\prime}} }} \left( {\theta (\varphi (u_{1} )),\theta (\varphi (u_{2} ))} \right).$

Thus, $\theta \circ \varphi$ is an isomorphism between ${\mathcal{G}}_{1}^{t}$ and ${\mathcal{G}}_{3}^{t} .$

Hence, it completes the proof.

## Complement of t-intuitionistic fuzzy graph

This section defines the concept of a complement of t-${\mathbb{IFG}}$ and investigates its essential features.

### Definition 23.

Let ${\mathcal{G}}_{1}^{t} = \left\langle {{\mathcal{A}}_{t} ,{\mathcal{B}}_{t} } \right\rangle$ be a t-${\mathbb{IFG}}$ of ${{\mathbb{G}}^{\prime}} = \left\langle {{\mathbb{V}},{\mathbb{E}}} \right\rangle$*.* The complement of a t-${\mathbb{IFG}}$ ${\mathcal{G}}_{1}^{t}$ is a t-${\mathbb{IFG}}$ $\overline{{{\mathcal{G}}_{1}^{t} }}$ on $\overline{{{{\mathbb{G}}^{\prime}}}} = \left\langle {{\overline{\mathbb{V}}},{\overline{\mathbb{E}}}} \right\rangle$ is defined as follows:

1. $${\overline{\mathbb{V}}} = {\mathbb{V}}$$
2. If $u_{1} \in {\mathbb{V}}$ then $\mu_{{\overline{{{\mathcal{A}}_{t} }} }} \left( {u_{1} } \right) = \mu_{{{\mathcal{A}}_{t} }} \left( {u_{1} } \right)$ and $\sigma_{{\overline{{{\mathcal{A}}_{t} }} }} \left( {u_{1} } \right) = \sigma_{{{\mathcal{A}}_{t} }} \left( {u_{1} } \right)$
3. If $\mu_{{{\mathcal{B}}_{t} }} \left( {u_{1} ,u_{2} } \right) \ne 0$ and $\sigma_{{{\mathcal{B}}_{t} }} \left( {u_{1} ,u_{2} } \right) \ne 0$ then $\mu_{{\overline{{{\mathcal{B}}_{t} }} }} \left( {u_{1} ,u_{2} } \right) = 0 = \sigma_{{\overline{{{\mathcal{B}}_{t} }} }} \left( {u_{1} ,u_{2} } \right)$
4. If $\mu_{{{\mathcal{B}}_{t} }} \left( {u_{1} ,u_{2} } \right) = 0 = \sigma_{{{\mathcal{B}}_{t} }} \left( {u_{1} ,u_{2} } \right) = 0$ then $\mu_{{\overline{{{\mathcal{B}}_{t} }} }} \left( {u_{1} ,u_{2} } \right) = min\left\{ {\mu_{{{\mathcal{A}}_{t} }} \left( {u_{1} } \right),\mu_{{{\mathcal{A}}_{t} }} \left( {u_{2} } \right)} \right\}$ and $\sigma_{{\overline{{{\mathcal{B}}_{t} }} }} \left( {u_{1} ,u_{2} } \right) = max\left\{ {\sigma_{{{\mathcal{A}}_{t} }} \left( {u_{1} } \right),\sigma_{{{\mathcal{A}}_{t} }} \left( {u_{2} } \right)} \right\}$

### Example 12.

**Figure 15:**
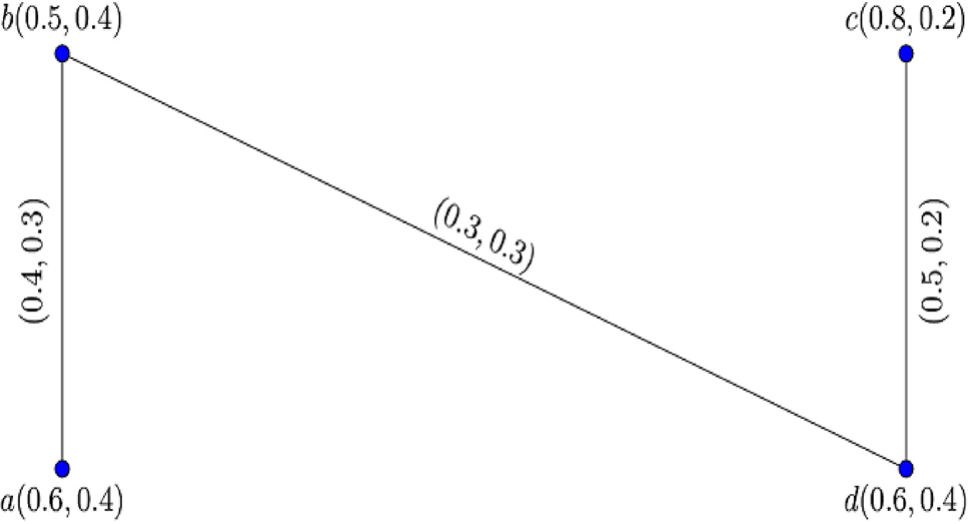
$$0.8 -$$$${\mathbb{IFG}}$$ $${\mathcal{G}}_{1}^{0.8}$$.

Consider a $0.8 - {\mathbb{IFG}}$ ${\mathcal{G}}_{1}^{t}$ as shown in Fig. 15.

**Figure 16:**
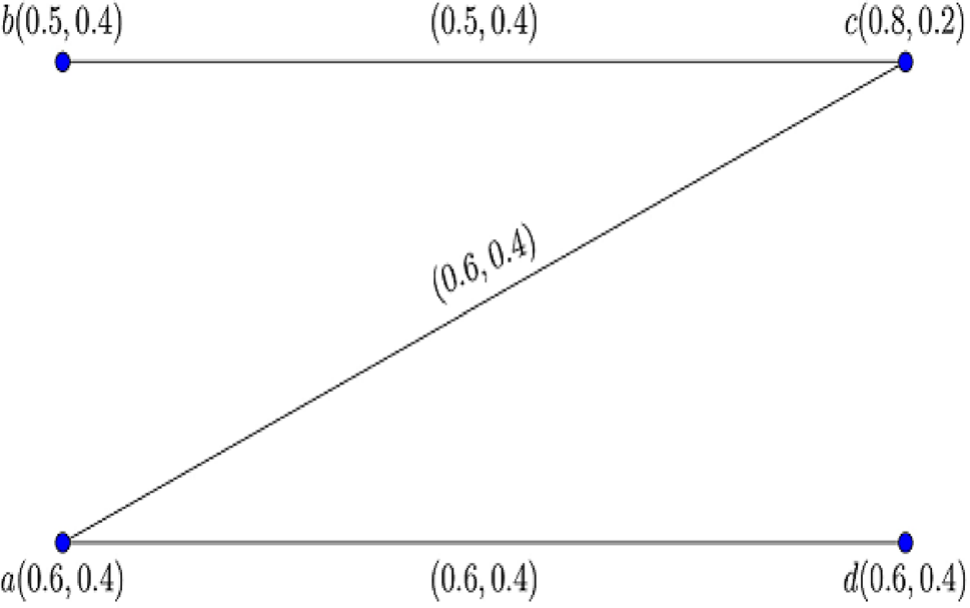
$$0.8 -$$$${\mathbb{IFG}}$$ $$\overline{{{\mathcal{G}}_{1}^{0.8} }}$$.

Then the complement $\overline{{{\mathcal{G}}_{1}^{t} }}$ of $0.8 - {\mathbb{IFG}}$ ${\mathcal{G}}_{1}^{t}$ is shown in Fig. 16.

### Definition 24.

A t-${\mathbb{IFG}}$ ${\mathcal{G}}_{1}^{t}$ is called self-complementary t-${\mathbb{IFG}}$ if $\overline{{{\mathcal{G}}_{1}^{t} }} \approx {\mathcal{G}}_{1}^{t} .$

### Proposition 7.

Let $${\mathcal{G}}_{1}^{t} = \left\langle {{\mathcal{A}}_{t} ,{\mathcal{B}}_{t} } \right\rangle$$ be a self-complementary t-$${\mathbb{IFG}}$$$$.$$ Then

$$\begin{aligned} & \mathop \sum \limits_{{u_{1} \ne u_{2} }} \mu_{{{\mathcal{B}}_{t} }} \left( {u_{1} ,u_{2} } \right) = \mathop \sum \limits_{{u_{1} \ne u_{2} }} min\left\{ {\mu_{{{\mathcal{A}}_{t} }} \left( {u_{1} } \right),\mu_{{{\mathcal{A}}_{t} }} \left( {u_{2} } \right)} \right\} \\ & \mathop \sum \limits_{{u_{1} \ne u_{2} }} \sigma_{{{\mathcal{B}}_{t} }} \left( {u_{1} ,u_{2} } \right) = \mathop \sum \limits_{{u_{1} \ne u_{2} }} max\left\{ {\sigma_{{{\mathcal{A}}_{t} }} \left( {u_{1} } \right),\sigma_{{{\mathcal{A}}_{t} }} \left( {u_{2} } \right)} \right\}. \\ \end{aligned}$$

### Proposition 8.

Let ${\mathcal{G}}_{1}^{t} = \left\langle {{\mathcal{A}}_{t} ,{\mathcal{B}}_{t} } \right\rangle$ be a t-${\mathbb{IFG}}$. If

$$\begin{aligned} & \mathop \sum \limits_{{u_{1} \ne u_{2} }} \mu_{{{\mathcal{B}}_{t} }} \left( {u_{1} ,u_{2} } \right) = \mathop \sum \limits_{{u_{1} \ne u_{2} }} min\left\{ {\mu_{{{\mathcal{A}}_{t} }} \left( {u_{1} } \right),\mu_{{{\mathcal{A}}_{t} }} \left( {u_{2} } \right)} \right\} \\ & \mathop \sum \limits_{{u_{1} \ne u_{2} }} \sigma_{{{\mathcal{B}}_{t} }} \left( {u_{1} ,u_{2} } \right) = \mathop \sum \limits_{{u_{1} \ne u_{2} }} max\left\{ {\sigma_{{{\mathcal{A}}_{t} }} \left( {u_{1} } \right),\sigma_{{{\mathcal{A}}_{t} }} \left( {u_{2} } \right)} \right\},\forall u_{1} ,u_{2} \in {\mathbb{V}} \\ \end{aligned}$$

Then $${\mathcal{G}}_{1}^{t}$$ is a self-complementary t-$${\mathbb{IFG}}$$$$.$$

### Proposition 9.

If ${\mathcal{G}}_{1}^{t}$ and ${\mathcal{G}}_{2}^{t}$ are two t-${\mathbb{IFG}}$ such that ${\mathbb{V}} \cap {{\mathbb{V}}^{\prime}} = \emptyset$ then $\overline{{{\mathcal{G}}_{1}^{t} + {\mathcal{G}}_{2}^{t} }} \cong \overline{{{\mathcal{G}}_{1}^{t} }} \cup \overline{{{\mathcal{G}}_{2}^{t} }} .$

### Proposition 10.

If ${\mathcal{G}}_{1}^{t}$ and ${\mathcal{G}}_{2}^{t}$ are two t-${\mathbb{IFG}}$ such that ${\mathbb{V}} \cap {{\mathbb{V}}^{\prime}} = \emptyset$ then $\overline{{{\mathcal{G}}_{1}^{t} \cup {\mathcal{G}}_{2}^{t} }} \cong \overline{{{\mathcal{G}}_{1}^{t} }} + \overline{{{\mathcal{G}}_{2}^{t} }} .$

### Proposition 11.

For any two t-${\mathbb{IFG}}$ ${\mathcal{G}}_{1}^{t}$ and ${\mathcal{G}}_{2}^{t} .$ If ${\mathcal{G}}_{1}^{t}$ and ${\mathcal{G}}_{2}^{t}$ have a strong isomorphism, then $\overline{{{\mathcal{G}}_{1}^{t} }}$ and $\overline{{{\mathcal{G}}_{2}^{t} }}$ also have a strong isomorphism.

### Proof.

Let $\varphi$ be a strong isomorphism between ${\mathcal{G}}_{1}^{t}$ and ${\mathcal{G}}_{2}^{t}$*.* Since $\varphi$ is a bijective map, then $\varphi^{ - 1}$ is also a bijective map such that $\varphi^{ - 1} \left( {w_{1} } \right) = u_{1}{_{1}} ,\forall q_{1} \in {{\mathbb{V}}^{\prime}}$. Thus.

$$\mu_{{{\mathcal{A}}_{t} }} \left( {\varphi^{ - 1} \left( {w_{1} } \right)} \right) = \mu_{{{\mathcal{A}}_{t}^{\prime} }} \left( {u_{1} } \right)\quad and\quad \sigma_{{{\mathcal{A}}_{t} }} \left( {\varphi^{ - 1} \left( {w_{1} } \right)} \right) = \sigma_{{{\mathcal{A}}_{t}^{\prime} }} \left( {u_{1} } \right),\forall w_{1} \in {{\mathbb{V}}^{\prime}}.$$

By employing Definition (23), it becomes evident that:

$$\begin{aligned} \mu_{{\overline{{{\mathcal{B}}_{t} }} }} \left( {u_{1} ,w_{1} } \right) = &\, min\left\{ {\mu_{{{\mathcal{A}}_{t} }} \left( {u_{1} } \right),\mu_{{{\mathcal{A}}_{t} }} \left( {w_{1} } \right)} \right\} \\ \le &\, min\left\{ {\mu_{{{\mathcal{A}}_{t}^{\prime} }} \left( {\varphi \left( {u_{2} } \right)} \right),\mu_{{{\mathcal{A}}_{t}^{\prime} }} \left( {\varphi \left( {w_{2} } \right)} \right)} \right\} \\ = &\, min\left\{ {\mu_{{{\mathcal{A}}_{t}^{\prime} }} \left( {u_{2} } \right),\mu_{{{\mathcal{A}}_{t}^{\prime} }} \left( {w_{2} } \right)} \right\} \\ = &\, \mu_{{\overline{{{\mathcal{B}}_{t}^{\prime} }} }} \left( {u_{2} ,w_{2} } \right) \\ \end{aligned}$$

Thus $$\mu_{{\overline{{{\mathcal{B}}_{t} }} }} \left( {u_{1} ,w_{1} } \right) \le \mu_{{\overline{{{\mathcal{B}}_{t}^{\prime} }} }} \left( {u_{2} ,w_{2} } \right)$$*.*

Also

$$\begin{aligned} \sigma_{{\overline{{{\mathcal{B}}_{t} }} }} \left( {u_{1} ,w_{1} } \right) = &\, max\left\{ {\sigma_{{{\mathcal{A}}_{t} }} \left( {u_{1} } \right),\sigma_{{{\mathcal{A}}_{t} }} \left( {w_{1} } \right)} \right\} \\ \le &\, \max \left\{ {\sigma_{{{\mathcal{A}}_{t}^{\prime} }} \left( {\varphi \left( {u_{2} } \right)} \right),\sigma_{{{\mathcal{A}}_{t}^{\prime} }} \left( {\varphi \left( {w_{2} } \right)} \right)} \right\} \\ = &\, \max \left\{ {\sigma_{{{\mathcal{A}}_{t}^{\prime} }} \left( {u_{2} } \right),\sigma_{{{\mathcal{A}}_{t}^{\prime} }} \left( {w_{2} } \right)} \right\} \\ = &\, \sigma_{{\overline{{{\mathcal{B}}_{t}^{\prime} }} }} \left( {u_{2} ,w_{2} } \right) \\ \end{aligned}$$

Consequently $$\sigma_{{\overline{{{\mathcal{B}}_{t} }} }} \left( {u_{1} ,w_{1} } \right) \le \sigma_{{\overline{{{\mathcal{B}}_{t}^{\prime} }} }} \left( {u_{2} ,w_{2} } \right)$$*.*

This shows that $\varphi^{ - 1}$ is a strong isomorphism between $\overline{{{\mathcal{G}}_{1}^{t} }}$ and $\overline{{{\mathcal{G}}_{2}^{t} }} .$

### Proposition 12.

Let $${\mathcal{G}}_{1}^{t}$$ and $${\mathcal{G}}_{2}^{t}$$ be two t-$${\mathbb{IFG}}$$$$.$$ Then $${\mathcal{G}}_{1}^{t} \approx {\mathcal{G}}_{2}^{t}$$ if and only if $$\overline{{{\mathcal{G}}_{1}^{t} }} \approx \overline{{{\mathcal{G}}_{2}^{t} }} .$$

### Proposition 13.

Let ${\mathcal{G}}_{1}^{t}$ and ${\mathcal{G}}_{2}^{t}$ be two t-${\mathbb{IFG}}$ $.$ If there is a co-strong isomorphism between ${\mathcal{G}}_{1}^{t}$ and ${\mathcal{G}}_{2}^{t} ,$ then there is a homomorphism between $\overline{{{\mathcal{G}}_{1}^{t} }}$ and $\overline{{{\mathcal{G}}_{2}^{t} }} .$

## Application of t-intuitionistic fuzzy graph

This section applies the theory of t-${\mathbb{IFG}}$ to the decision-making process of alleviating poverty*.*

**Figure 17:**
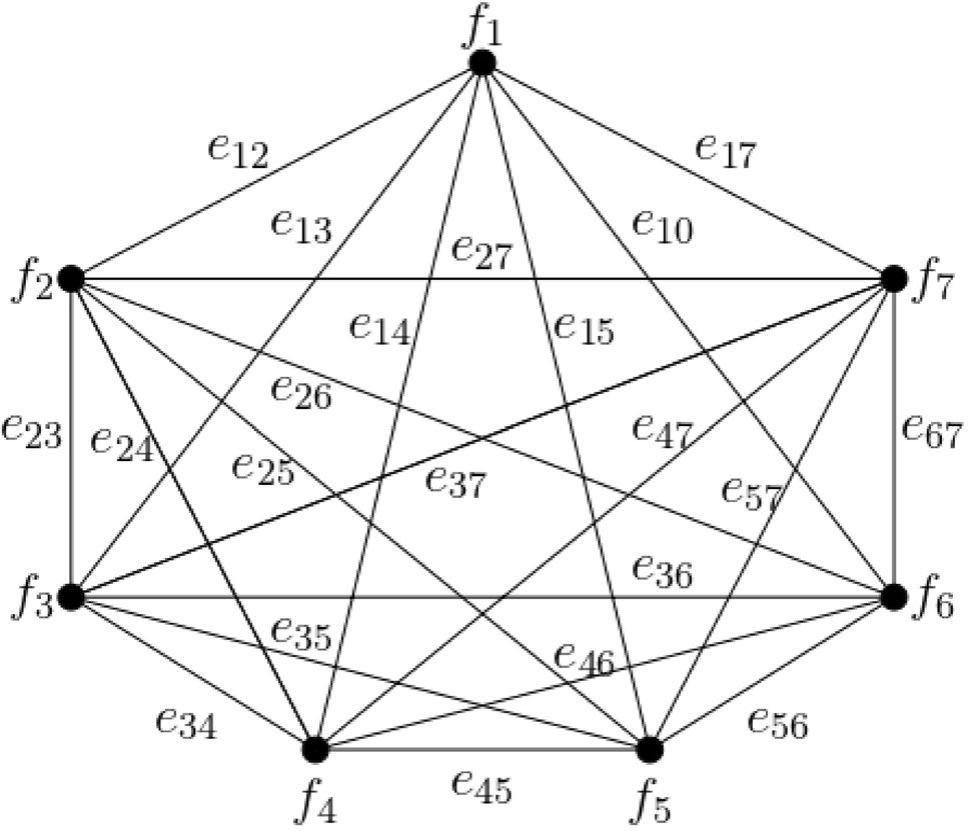
Graphical representation of poverty reduction factors.

Developing nations have been profoundly affected by extreme destitution, which has significantly impacted their economies, societies, and a vast number of people globally. The escalation in the poverty rate can be attributed to various factors. Poverty is characterized by the inability to provide oneself and one's family with necessities such as food, clothing, and shelter. It can be examined from psychological, social, political, and economic perspectives. These circumstances can lead to criminal activity, drug abuse, and even fatalities. To address poverty reduction effectively, the t-${\mathbb{IFG}}$ provides a mathematical representation and analysis of uncertain data. By utilizing the t-${\mathbb{IFG}}$, we can model and analyze elements related to poverty alleviation. This approach enables us to identify the most crucial variables in systematically and organized eliminating poverty, enhancing decision-making in poverty reduction efforts. Reducing poverty requires a multifaceted strategy that addresses the underlying causes of poverty and implements interventions designed to alleviate it. Some main factors are beneficial in reducing poverty, such as promoting economic growth $\left( {f_{1} } \right),$ creating employment opportunities $(f_{2} ),$ enhancing access to education and skills training $(f_{3} ),$ promoting manufacturing sectors $(f_{4} ),$ promoting industrialization $(f_{5} ),$ improving agriculture $(f_{6} ),$ and improving infrastructure $(f_{7} ).$ Let ${\mathbb{V}} = \left\{ {f_{1} ,f_{2} ,f_{3} ,f_{4} ,f_{5} ,f_{6} ,f_{7} } \right\}$ represents the vertex of the set of factors that significantly contribute to the fight against poverty. Let the edges depict the degree of connection or relationship between the factors as t-intuitionistic fuzzy values. The graphical representation of the factor of reduction poverty is displayed in Fig. 17. Within the poverty reduction framework, membership and non-membership functions in intuitionistic fuzzy logic denote the connection between two distinct items or factors. These functions capture the degree to which an element exhibits membership or non-membership in a particular factor, facilitating a complete understanding of their interconnections. Within the poverty reduction framework, the membership function concept pertains to the extent to which an element exhibits favorable alignment with a particular factor. The non-membership function is a measure that measures the extent to which an element deviates from or lacks affiliation with a particular factor. The integration of these two functions offers a comprehensive understanding of the correlation between an element and a particular factor, encompassing its positive correlation and divergence from said factor. Decision-makers can evaluate the intricate and uncertain connections between different aspects of poverty reduction efforts by considering membership and non-membership functions. The parameter 't' allows decision-makers to customize the t-${\mathbb{IFG}}$ according to their domain knowledge and problem.

Moreover, different parameter values of 't' indicate different attitudes towards risk and uncertainty. The direction of threshold values for membership and non-membership allows decision-makers to highlight or de-emphasize certain facts depending on their desired degree of membership and non-membership. The parameter denoted as 't' enables the adaptation of t-${\mathbb{IFG}}$ to different contexts and sensitivities. Adjusting variable 't' allows decision-makers to explore various possibilities by manipulating the balance between positive and opposing viewpoints. This ability is critical when making decisions in uncertain contexts or during ongoing changes.

The $${\mathbb{IFS}}$$ and $$0.8 -$$$${\mathbb{IFS}}$$ defined on the edges is shown in the following Table 1.

**Table 1:** Edges of ${\mathbb{IFS}}$ and $0.8 - {\mathbb{IFS}}$.

Edges	${\mathbb{IFS}}$	0.8-${\mathbb{IFS}}$	Edges	${\mathbb{IFS}}$	0.8-${\mathbb{IFS}}$
$e_{12} = \left( {f_{1} ,f_{2} } \right)$	$\left( {0.9,0.1} \right)$	$\left( {0.8,0.2} \right)$	$e_{34} = \left( {f_{3} ,f_{4} } \right)$	$\left( {0.9,0.1} \right)$	$\left( {0.8,0.2} \right)$
$e_{13} = \left( {f_{1} ,f_{3} } \right)$	$\left( {0.7,0.3} \right)$	$\left( {0.7,0.3} \right)$	$e_{35} = \left( {f_{3} ,f_{5} } \right)$	$\left( {0.8,0.2} \right)$	$\left( {0.6,0.4} \right)$
$e_{14} = \left( {f_{1} ,f_{4} } \right)$	$\left( {0.8,0.2} \right)$	$\left( {0.8,0.2} \right)$	$e_{36} = \left( {f_{3} ,f_{6} } \right)$	$\left( {0.7,0.3} \right)$	$\left( {0.7,0.3} \right)$
$e_{15} = \left( {f_{1} ,f_{5} } \right)$	$\left( {0.5,0.5} \right)$	$\left( {0.5,0.5} \right)$	$e_{37} = \left( {f_{3} ,f_{7} } \right)$	$\left( {0.6,0.4} \right)$	$\left( {0.5,0.5} \right)$
$e_{16} = \left( {f_{1} ,f_{6} } \right)$	$\left( {0.9,0.1} \right)$	$\left( {0.8,0.2} \right)$	$e_{45} = \left( {f_{4} ,f_{5} } \right)$	$\left( {0.4,0.4} \right)$	$\left( {0.4,0.4} \right)$
$e_{17} = \left( {f_{1} ,f_{7} } \right)$	$\left( {0.7,0.3} \right)$	$\left( {0.7,0.3} \right)$	$e_{46} = \left( {f_{4} ,f_{6} } \right)$	$\left( {0.3,0.4} \right)$	$\left( {0.3,0.4} \right)$
$e_{23} = \left( {f_{2} ,f_{3} } \right)$	$\left( {0.5,0.4} \right)$	$\left( {0.5,0.4} \right)$	$e_{47} = \left( {f_{4} ,f_{7} } \right)$	$\left( {0.7,0.3} \right)$	$\left( {0.7,0.3} \right)$
$e_{24} = \left( {f_{2} ,f_{4} } \right)$	$\left( {0.6,0.4} \right)$	$\left( {0.6,0.4} \right)$	$e_{56} = \left( {f_{5} ,f_{6} } \right)$	$\left( {0.5,0.5} \right)$	$\left( {0.5,0.5} \right)$
$e_{25} = \left( {f_{2} ,f_{5} } \right)$	$\left( {0.7,0.3} \right)$	$\left( {0.7,0.3} \right)$	$e_{57} = \left( {f_{5} ,f_{7} } \right)$	$\left( {0.9,0.1} \right)$	$\left( {0.8,0.2} \right)$
$e_{26} = \left( {f_{2} ,f_{6} } \right)$	$\left( {0.5,0.5} \right)$	$\left( {0.5,0.5} \right)$	$e_{67} = \left( {f_{6} ,f_{7} } \right)$	$\left( {0.6,0.4} \right)$	$\left( {0.6,0.4} \right)$
$e_{27} = \left( {f_{2} ,f_{7} } \right)$	$\left( {0.5,0.5} \right)$	$\left( {0.5,0.5} \right)$			

In Table 1, the edge $e_{12}$ from “promoting economic growth” to "creating employment opportunities" indicates that promoting economic growth is related to creating more job opportunities. In edge $e_{12} = \left( {0.8,0.2} \right)$, the membership degree 0.8 indicates a strong connection between these factors, and the non-membership degree 0.2 shows a weak connection between these factors in reducing poverty. In the same way, an edge $e_{36}$ from "improving access to education and skills training" to "improving agriculture" shows that better education and skills training can lead to better farming practices and higher productivity. In the given context, the membership degree of 0.7 for the edge $e_{36} = \left( {0.7,0.3} \right)$ signifies a significant correlation or positive influence between the factors to reduce poverty. Conversely, the non-membership degree of 0.3 suggests a relatively low perception of disassociation or lack of relevance between these factors and poverty reduction efforts. Here, the parameter “*t*” suggests which factor can reduce poverty by $80\%$*.*

As shown in Table 2, the application of part $\left( 1 \right)$ of Definition (10) yields the following results.

**Table 2:** Table of membership and non-membership degree of each factor.

Factors	Degree of each factor
$f_{1}$	$\deg \left( {f_{1} } \right) = \left( {4.3,1.7} \right)$
$f_{2}$	$\deg \left( {f_{2} } \right) = \left( {3.6,2.3} \right)$
$f_{3}$	$\deg \left( {f_{3} } \right) = \left( {3.8,2.1} \right)$
$f_{4}$	$\deg \left( {f_{4} } \right) = \left( {3.6,1.9} \right)$
$f_{5}$	$\deg \left( {f_{5} } \right) = \left( {3.5,2.3} \right)$
$f_{6}$	$\deg \left( {f_{6} } \right) = \left( {3.4,2.3} \right)$
$f_{7}$	$\deg \left( {f_{7} } \right) = \left( {3.8,2.2} \right)$

The score function of the edges is defined as:

$${\mathfrak{T}} = \sqrt {\mu_{j}^{2} + \left( {1 - \sigma_{j} } \right)^{2} } ,1 \le j \le 7$$

The score function of the edges is calculated to find the optimal factor.

The results shown in Table 3 are then obtained by using the score function formula from Table 2.

**Table 3:** Score value of each factor.

Factors	Score function ${\mathbf{\mathfrak{T}}}\left( {{\varvec{f}}_{{\varvec{j}}} } \right)$
$f_{1}$	4.3566
$f_{2}$	3.8275
$f_{3}$	3.9560
$f_{4}$	3.7108
$f_{5}$	3.7336
$f_{6}$	3.6401
$f_{7}$	3.9850

**Figure 18:**
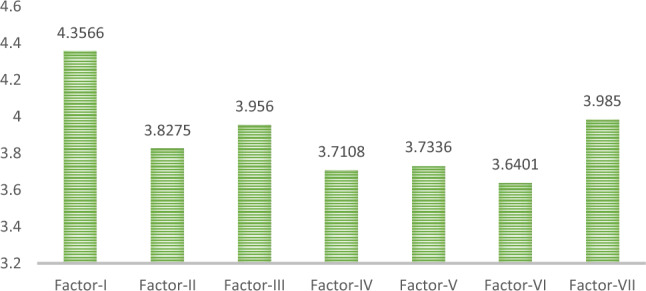
Graphical representation of score function of factor.

Figure 18 depicts the graphical representation of the score function for the factors listed in Table 3*.*

Consequently, ${\mathfrak{T}}\left( {f_{1} } \right) = 4.3566$ is the greatest value, and according to the parameter $t$*,* $f_{1}$ is the most significant factor in reducing poverty. Promoting economic growth can create jobs, raise incomes, encourage entrepreneurship and new ideas, lower the prices of goods and services, and give governments more money to spend on social services and programs. All of these things can help reduce poverty.

## Comparative analysis

The t-${\mathbb{IFG}}$ is an improved variant of the intuitionistic fuzzy graph that includes an additional parameter referred to as $t,t \in \left[ {0,1} \right].$ By adjusting the ‘*t*’ parameter, uncertainty modeling can be fine-tuned to fit specific requirements and domain characteristics better. By changing the value of the parameter $t$ various decision-making or preference scenarios can be depicted, providing a more precise representation of uncertainty and vagueness. The t-${\mathbb{IFG}}$ offer various applications in diverse situations and decision-making processes. Their adjustable parameter ‘*t*’ within the closed unit interval enables the capture of varying degrees of conservatism or optimism, allowing for customization according to specific requirements. This notion is beneficial in problem-solving domains where multiple levels of uncertainty, hesitancy, and decision preferences must be considered simultaneously. Their effectiveness shines in complex decision-making scenarios, including medical diagnosis, pattern recognition, and decision support systems, as they can accommodate different levels of uncertainty and hesitancy. The exceptional flexibility and adaptability of t-${\mathbb{IFG}}$ make them the preferred choice when a more precise representation of uncertainty is necessary.

Furthermore, when the parameter $t$ is assigned a value of $0.1$ within the framework of utilizing t-intuitionistic fuzzy sets to tackle the problem of poverty reduction, it signifies a prudent and somewhat negative assessment of the effectiveness of different factors in alleviating poverty. A membership degree of $0.1$ indicates a weak association between the variables, implying that the impact of poverty reduction is limited. On the other hand, a non-membership degree of $0.9$ signifies a perceived lack of a robust correlation or a fragile link between these variables and the mitigation of poverty. When the degrees of membership and non-membership stay consistent, the elements under examination possess a uniform and equivalent amount of association with a certain factor and a consistent level of non-association. The observed uniformity indicates that all aspects are seen as equally connected to the factor in question, without any noticeable differentiation based on their levels of membership or non-membership. The constancy of ambiguity or reluctance in associating these elements with the factor persists uniformly across all dimensions. Choosing a parameter value of 't' near zero signifies a need for more precision about the impacts on poverty alleviation.

## Conclusion

In this research, the concept of t-intuitionistic fuzzy graphs (t-${\mathbb{IFG}}$) has been initiated, and various fundamental features of this phenomenon have been explored. Many set-theoretical operations of t-${\mathbb{IFG}}$ have been studied, and graphical representations of these operations have been demonstrated. Additionally, the idea of a complement of t-${\mathbb{IFG}}$ has been defined, and some of its key features have been investigated. The notions of homomorphisms and isomorphisms of t-${\mathbb{IFG}}$ have been introduced. Furthermore, a practical application of the newly defined technique in reducing poverty has been presented.

The use of t-${\mathbb{IFG}}$ effectively addresses real-world problems and improves decision-making processes. It is a flexible and robust framework that deals with imprecision and uncertainty in decision-making while optimizing complex systems, recognizing patterns, and offering various applications for computational intelligence. This idea has the potential for future use in healthcare systems, transportation networks, pattern recognition, and machine learning.

Selecting a parameter value 't' close to zero indicates a lack of identifiable specificity in the effects of poverty reduction. In contrast, when the parameter value 't' approaches 1, it strongly signifies a robust and visible correlation with achieving objectives related to reducing poverty. In t-${\mathbb{IFG}}$, the parameter 't' measures the level of assurance or uncertainty over the effectiveness of poverty reduction efforts. The extremes of this parameter indicate either a negligible impact or a strong correlation with the desired outcome. Utilizing this calibrated parameter allows decision-makers to precisely adjust the depiction of uncertainty and its influence on analytical results, leading to a sophisticated and flexible structure for tackling the intricate complications of poverty reduction.

One of our primary goals for future studies is to apply the proposed strategy to solve MCDM problems, specifically supplier selection, risk management, and renewable energy selection. The proposed techniques will also be applied to neural networks, clustering, feature selection, and risk management. In addition, some advanced decision-making techniques of complex spherical fuzzy Aczel Alsina aggregation operators^61^ will also be studied within the context of the strategies presented in this article.

## Data Availability

All data generated or analyzed during this study are included in this published article.
